# Cofilin/RhoA and CREB synergistically promote invasion and migration of polyploid giant cancer cells in colorectal cancer

**DOI:** 10.7150/jca.138960

**Published:** 2026-09-03

**Authors:** Yidi Ning, Kai Liu, Jing Xu, Liechen Ji, Shiwu Zhang

**Affiliations:** 1 Nankai University School of Medicine. Tianjin, 300071, P. R. China.; 2 Department of Research and Education, Tianjin Beichen Hospital, Tianjin, 300499 P.R. China.; 3 Department of General Surgery, Tianjin Union Medical Center, the First Affiliated Hospital of Nankai University, Tianjin, 300121, P. R. China.; 4 Department of colorectal surgery, Tianjin Union Medical Center, the First Affiliated Hospital of Nankai University Tianjin, 300121, P. R. China.; 5 Department of pathology, Tianjin Union Medical Center, the First Affiliated Hospital of Nankai University, Tianjin, 300121, P. R. China.

**Keywords:** Colorectal cancer, Polyploid giant cancer cells, Cofilin, SUMOylation

## Abstract

Colorectal cancer (CRC) is associated with high mortality and poor survival. Polyploid giant cancer cells (PGCCs) are a subset of cancer cells that drive tumor initiation and progression. In this study, high-concentration CoCl₂ treatment was used to induce Hct116 and LoVo cells to form PGCCs with daughter cells (PDCs), which exhibited enhanced invasion and migration capabilities. Cofilin expression was elevated in PDCs compared to that in the control cells. Mechanistically, the small ubiquitin-like modification (SUMOylation) of cofilin promoted its nuclear translocation and contributed to the aggressive phenotype of PDCs, with lysine 132 being identified as the key modification site mediated by tripartite motif-containing 28 (TRIM28). In addition, TRIM28 directly interacted with cofilin and inhibited its degradation, thereby increasing its stability. Nuclear cofilin facilitated RNA polymerase II-dependent upregulation of Ras homolog family member A (RhoA) transcription. RhoA enhanced nuclear accumulation of phosphorylated CREB (p-CREB), which further promoted cofilin transcription by binding to its promoter region. Cofilin knockdown inhibited the growth of xenograft tumors derived from Hct116 and LoVo PDCs. Regression analysis revealed that cofilin overexpression was associated with aggressive clinicopathological features in patients with CRC, although it did not significantly correlate with overall survival. Collectively, these findings revealed that cofilin is regulated by TRIM28-mediated SUMOylation and suggested that targeting the cofilin/RhoA/CREB axis may represent a promising therapeutic strategy for CRC.

## Introduction

Colorectal cancer (CRC) has shown a steady increase in incidence and mortality in recent years, making it the third most common cancer and a major threat to human health 1, 2. Somatic mutations, epigenetic alterations, and the host immune system are key factors that regulate tumor development 3-5. Surgery remains the preferred first-line treatment for CRC and is often combined with chemotherapy and radiotherapy. Immunotherapy, targeted therapy, and nanotherapy have emerged as novel therapeutic strategies. Despite advances in CRC diagnosis and treatment, patient prognosis remains poor 6. Most tumor recurrences occur as metastases, which are the leading cause of death among patients with CRC 7, 8.

Intratumor heterogeneity is a fundamental driver of cancer progression and metastasis 9. Recently, a subpopulation of cancer cells characterized by highly enlarged or multiple nuclei, often accompanied by increased cell size and disordered genomic content, has been identified. These are known as polyploid giant cancer cells (PGCCs) 10-12. Drug resistance often arises when tumor cells acquire genomic alterations such as mutations, amplifications, or deletions that impair downstream effector activation and signal transduction 13. PGCCs are resistant to radiotherapy and chemotherapy. Under microenvironmental stress, these cells maintain a relatively quiescent cell cycle. PGCCs can be induced by various factors, including nutrient deprivation, hypoxia, and antitumor therapies 14, 15. When provided with adequate nutrients and oxygen in culture, PGCCs exit dormancy and generate daughter cells through asymmetric division processes, such as splitting, budding, or bursting, thereby contributing to tumor aggressiveness and progression 16, 17.

Actin filaments are key cytoskeletal components, and their remodeling provides the structural basis and driving force for morphological changes, invasion, and migration of tumor cells. The actin-depolymerization factor (ADF)/cofilin family of actin-binding proteins plays a key role in regulating actin filament dynamics 18. The ADF family includes cofilin 1 and cofilin 2. Studies have shown that cofilin 1 (hereafter referred to as cofilin) is highly expressed in several cancers, including ovarian, liver, and colon cancers. Evidence suggests that cofilin depolymerizes actin filaments, accelerates actin turnover, and alters the invasive properties of tumor cells 19-21. Therapeutic agents are influenced by cellular metabolism, and abnormal levels of cyclic adenosine monophosphate (cAMP) are associated with increased apoptosis and treatment resistance. Actin polymerization is regulated by protein kinase A (PKA) and PKC 22. The cAMP response element-binding protein (CREB), a downstream effector of cAMP and PKA signaling, also participates in this process 23. Actin cytoskeletal dynamics depend on PKA to modulate adhesion-associated events 24. Therefore, investigating the mechanistic interactions between the PKA/CREB pathway and cofilin is critical for elucidating their functions.

Post-translational modifications are critical for regulating cellular homeostasis and adaptive responses to stimuli 25, 26. Dysregulated modifications are prevalent in cancer and play important roles in disease pathogenesis 27. Small ubiquitin-like modifier (SUMO) and ubiquitin proteins share similar molecular structures and mechanisms. Although the binding of ubiquitin to substrate proteins typically triggers their degradation, SUMOylation is covalently conjugated to the lysine residues of substrates, thereby influencing their subcellular localization, stability, and enzyme activity 28. Both SUMO1 and ubiquitin bind to the lysine residues on their substrates, leading to competitive inhibition. Consequently, SUMOylation machinery components are subject to crosstalk with ubiquitination, which can alter protein levels or activity 29. In this study, we demonstrated that PGCCs with daughter cells (PDCs) exhibited higher proliferation, migration, and invasion capabilities than control cells. Cofilin overexpression promoted epithelial-mesenchymal transition (EMT), migration, and invasion in Hct116 and LoVo PDCs. Mechanistically, SUMOylation of cofilin promoted its nuclear translocation, which enhanced the recruitment of RNA polymerase II to the Ras homolog family member A (RhoA) promoter, thereby upregulating the expression of phosphorylated CREB (p-CREB) in the nucleus. Furthermore, we found that SUMOylation of cofilin at lysine 132 (K132) was mediated by the E3 SUMO ligase tripartite motif-containing 28 (TRIM28), a process that enhanced cofilin stability. Collectively, these findings indicate that the cofilin/RhoA/CREB feedback loop is involved in the migration and invasion of CRC cells.

## Materials and Methods

*Cell culture and formation of PGCCs*. Human CRC cell lines, Hct116 and LoVo, were purchased from the American Type Culture Collection (ATCC, USA) and tested for *mycoplasma* contamination. PGCCs were induced as described previously by our group 10, 17. Briefly, cells were cultured in complete Roswell Park Memorial Institute 1640 medium supplemented with 10% fetal bovine serum and 1% penicillin-streptomycin and maintained at 37 ℃ in a humidified incubator with 5% CO_2_. Upon reaching approximately 70%-80% confluence, CRC cells were exposed to 450 μM cobalt chloride (CoCl_2_) for 48 hours. Following a 2-3**-**week recovery period in complete 1640 medium, the resulting PGCCs and their daughter cells were used in subsequent experiments.

*Western blotting (WB)*. For protein extraction, cells were lysed in radioimmunoprecipitation assay buffer (Solarbio, R0010) or using a nuclear and cytoplasmic protein extraction kit (Abbkine, KTP3001), and the supernatant was collected. For co-immunoprecipitation (co-IP), cells were lysed in IP buffer (Abbkine, KTP3001). The supernatant was incubated overnight with protein A/G agarose beads and primary antibodies. The immunoprecipitates were eluted with 2× loading buffer. Proteins were separated by sodium dodecyl sulfate-polyacrylamide gel electrophoresis or non-reducing gel electrophoresis and then transferred onto a polyvinylidene difluoride membrane. Membranes were incubated with primary antibodies, followed by enhanced chemiluminescence detection. Protein expression levels were quantified by measuring the band intensities for statistical analysis. Details of the antibodies and reagents used are listed in Table S1. For the endogenous SUMOylation analysis, cofilin was extracted and immunoprecipitated using NEM-containing lysis buffer, followed by non-reducing gel electrophoresis.

*RNA interference, plasmid transfection, and lentiviral infection*. Targeting knockdown specific genes using small interfering RNA (siRNA) oligonucleotides, which were scheduled and produced by Genema (Nanjing, China), and the siRNA sequences are listed in Table S2. The most effective siRNA (si 2#) targeting cofilin was selected, and the corresponding lentiviral shRNA plasmid was purchased from GeneChem (Shanghai, China). The mutant plasmids were constructed by GenCefe Biotech Co., Ltd. (Wuxi, China). shRNA-expressing cells were selected using puromycin (MCE, HY-B1743) until stable knockdown of cofilin expression was achieved.

*Quantitative real-time polymerase chain reaction (qRT-PCR)*. The primer sequences are listed in Table S3. Total RNA was extracted using the TRIzol reagent, isopropanol, and trichloromethane. Reverse transcription was performed using 4×Hifair® Ⅲ SuperMix (YEASEN, 11137). Polymerase chain reaction amplification was conducted using specific primers on a thermocycler (Thermo Fisher Scientific, USA). The fold change (2^-ΔΔCt^) was used to analyze mRNA expression levels.

*Colony formation, wound-healing, and Transwell assays*. For the colony formation assay, treated cells were cultured until visible colonies formed. Colonies were counted using ImageJ software. For the wound-healing assay, treated cells were seeded in six-well plates, and a standardized scratch was introduced. Cell invasion ability was calculated as: Invasion rate= (1-(24-h wound area / 0-h wound area)) × 100%. For Transwell invasion and migration assays, cells were placed in the upper chamber, which has a filter membrane on its underside. Invading or migrating cells on the membranes were imaged and counted in three random visual fields to assess cell invasion and migration ability.

*Cell counting kit-8 (CCK-8) assay*. Hct116 and LoVo PDCs were seeded into 96-well transparent plates and treated with inhibitors or activators (Table S4) for the indicated time periods. Subsequently, the CCK-8 solution (Beyotime, C0038) was added to each well, and the absorbance and cell viability was measured at 450 nm.

*Immunofluorescence (IF) and immunocytochemical staining.* The cells seeded on coverslips were fixed with ice-cold 4% paraformaldehyde. For immunofluorescence, the samples were incubated with primary antibodies or phalloidin, followed by Alexa Fluor-conjugated secondary antibodies. Cell nuclei were counterstained with 4′,6-diamidino-2-phenylindole (DAPI; Solarbio, C0065). Immunocytochemical analysis was performed according to the manufacturer's instructions (ZSGB-BIO, SPN-9002). Briefly, the cells were incubated with primary antibodies, followed by staining with DAB and hematoxylin. Images were captured using a Nikon fluorescence microscope and an Olympus confocal microscope.

*Cell cycle and apoptosis assays*. Approximately 1 × 10^6^ cells were fixed with 70% ice-cold ethanol for several hours, and stained with propidium iodide (PI; Beyotime, C1052) in the dark, followed by flow cytometry (Agilent, USA). For apoptosis assays, treated cells were stained with Annexin V-FITC and propidium iodide and analyzed by fluorescence microscopy (Nikon) or flow cytometry.

*Chromatin immunoprecipitation (ChIP).* The assay was performed according to the manufacturer's instructions using a ChIP kit (Absin, abs50034). Cells were incubated with 1% formaldehyde to cross-link the DNA-protein complexes, and the reaction was terminated with a glycine buffer. Cells were then lysed, and chromatin was fragmented by sonication. Protein A/G beads and primary antibodies were added to the DNA extract for immunoprecipitation. The resulting immunoprecipitates were analyzed by WB and PCR.

*Dual-luciferase reporter assay*. Cells were co-transfected with different constructs: pGL4.73 (Renilla luciferase vector), pGL3-Basic (firefly luciferase reporter vector) containing cofilin (CFL)-specific promoter regions, and pcDNA3.1 carrying wild-type CREB. Firefly and Renilla luciferase activities were measured by detecting the absorbance at 480 and 520 nm, respectively. Firefly luciferase activity was normalized to Renilla luciferase activity.

*Xenograft animal assay*. The animal study was approved by the Hospital Review Board of Tianjin People's Hospital. Thirty BALB/c nude mice (4-6 weeks old) were used to assess the tumorigenesis of cofilin-knockdown PDCs. They were obtained from Vital River Co., Ltd. (Beijing, China) and subcutaneously injected with 5 × 10⁶ cells into the inguinal region, including groups injected with Hct116 and LoVo control cells, sh-NC PDCs, and sh-cofilin PDCs (randomly assigned five mice per group). Mice were housed under controlled conditions (21 ± 2 °C; humidity: 50 ± 10%). Tumor volumes were measured every 4 days post-injection. The tumor volume was calculated by length × width^2^ × 0.5 (cm^3^). Several days after the injection, the mice were euthanized by cervical dislocation, and the xenografted tumors were harvested.

*Tissue samples*. A total of 119 tissue samples from CRC patients were used to construct a tissue microarray. The samples were stratified by histological grade into 77 well-differentiated and 42 moderately or poorly differentiated tumors. Table S5 presents the results of logistic and Cox regression analyses of clinicopathological characteristics and cofilin immunohistochemistry (IHC) scores. All procedures were approved by the Hospital Review Board of Tianjin Union Medical Center.

*Immunohistochemical staining*. Tumor tissues were fixed in formalin, embedded in paraffin, sectioned, deparaffinized, and rehydrated in graded ethanol solutions. Antigen retrieval was performed using a citrate buffer, after which the sections were incubated with primary antibodies and stained using an IHC kit (ZSGB-BIO, PV9000). The percentage of positive staining was graded as 1 (< 25%), 2 (25-50%), or 3 (> 50%). The staining intensity was scored as 0 (negative), 1 (weakly positive), 2 (moderately positive), or 3 (strongly positive). The final score was calculated by multiplying the percentage grade by the intensity score. Samples with a final score ≤ 3 were classified as a low-expression group. Tissue imaging was performed using a Nikon microscope.

*Database analyses*. Gene expression data were obtained from GEPIA, which utilizes RNA-seq data from the TCGA and GTEx projects 30. The infer correlations between cofilin expression and tumor-infiltrating immune cell abundance using TISIDB database 31.

*Statistical analyses.* All statistical analyses were performed using GraphPad Prism 9 software. Differences between the two groups were determined using an unpaired two-tailed Welch's t-test, and differences among multiple groups were analyzed using the Kruskal-Wallis test with a post-hoc correction. Data were presented as the mean ± standard deviation. An adjusted *P-*value < 0.05 was considered statistically significant.

## Results

**Figure 1:**
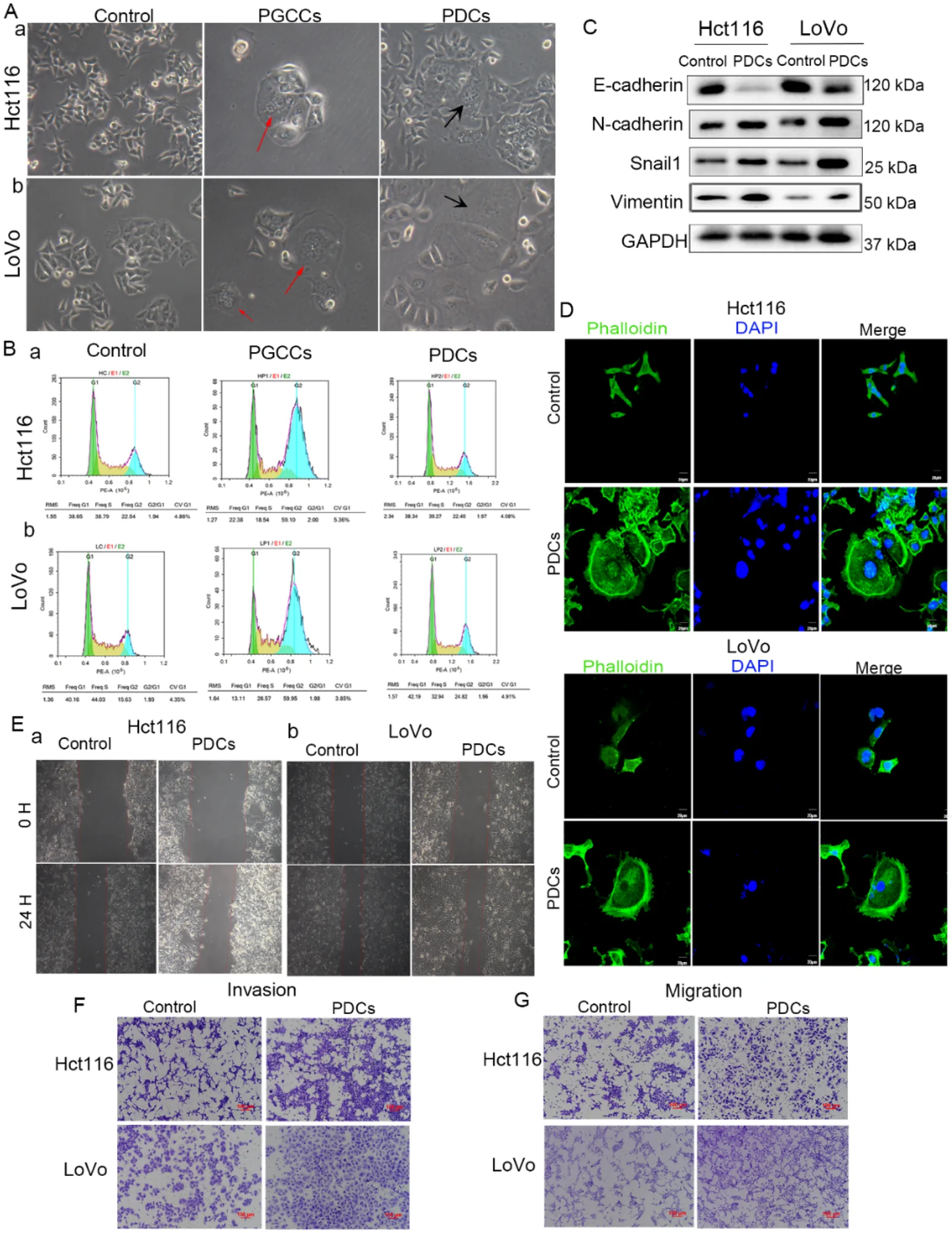
** PDCs formation following CoCl₂ treatment.** A. Hct116 and LoVo cells were treated with 450 μM CoCl_2_ to induce PGCCs and PDCs (red arrowheads, PGCCs; black arrowheads, PGCCs with daughter cells). B. Cell cycle analysis of Hct116 and LoVo control cells, PGCCs and PDCs. C. WB analysis of EMT protein expression in Hct116 and LoVo control cells and PDCs. D. IF staining of F-actin in Hct116 and LoVo control cells and PDCs using phalloidin. E. Wound healing assays assessing the invasive ability of control cells versus PDCs in (a) Hct116 and (b) LoVo cells. F. Transwell invasion assay and G. Transwell migration assay showing invasion and migration capabilities of Hct116 and LoVo control cells and PDCs.

*Formation of PGCCs and PDCs following CoCl₂ treatment.* CoCl₂ is a well-established chemical hypoxia mimetic widely used in hypoxia-related disease research. Hct116 and LoVo cells were exposed to a high concentration (450 μM) of CoCl_2_ for 48 hours, after which the culture medium was withdrawn. Although most cells gradually died following treatment, the remaining cells were allowed to recover in fresh complete medium. Cytotoxicity was assessed using the CCK-8 assay, which revealed a marked reduction in cell viability during the initial 2-day treatment period, followed by increased proliferation from days 3 to 10. By the end of this period, cell viability had recovered to levels comparable to those of the untreated tumor cells (Fig. S1). After approximately 14 days of recovery from CoCl₂ exposure, the surviving cells flattened and exhibited progressive cytoplasmic expansion and/or an increased number of nuclei. Notably, the PGCCs were at least three-fold larger than normal Hct116 and LoVo cells and displayed polyploid genomes. Over the subsequent 3-4 days, PGCCs underwent asymmetric division, generating numerous diploid daughter cells. PGCCs containing multiple daughter cells were designated PDCs (Figure 1A). Cell cycle analysis revealed an altered distribution of Hct116 and LoVo PGCCs, with an increased proportion of cells in the G2 phase, whereas PDCs exhibited G2-phase fractions comparable to those of normal cancer cells (Figure 1B). To obtain sufficient PDCs for subsequent experiments, the cells were subjected to multiple rounds of CoCl₂ treatment.

To evaluate the invasive and migratory capabilities of PDCs, WB was performed to detect the expression of EMT-related proteins. The results showed that E-cadherin was downregulated, whereas N-cadherin, Snail1, and vimentin were upregulated in PDCs (Figure 1C, Fig. S1). Immunofluorescence (IF) staining of F-actin with phalloidin revealed more intense F-actin filaments in PDCs than in the control Hct116 and LoVo cells. Additionally, the PDCs exhibited a greater number of filamentous protrusions along the cell periphery (Figure 1D). Wound healing assays (Figure 1E, Fig. S1) and Transwell invasion and migration assays demonstrated that PDCs possessed significantly higher invasive and migratory capacities than control cells (Figure 1F-1G, Fig. S1). Collectively, these findings indicate that CoCl₂-induced PGCC-derived PDCs acquired EMT-like properties, resulting in a more aggressive and invasive phenotype in the Hct116 and LoVo cell lines.

**Figure 2:**
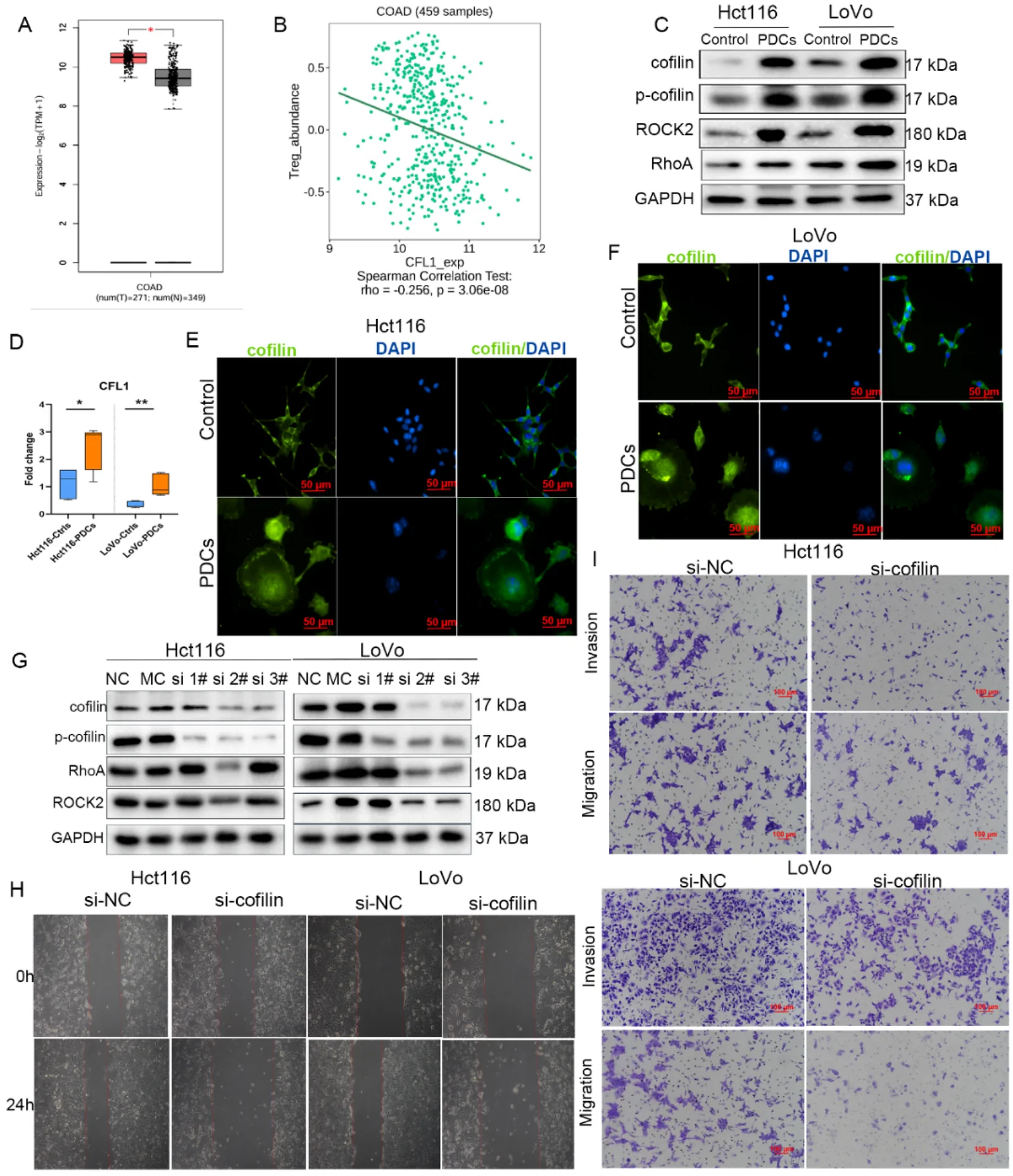
** Overexpression of cofilin regulates the RhoA pathway to promote invasion and migration of PDCs.** A. GEPIA database analysis reveals cofilin overexpression in COAD. B. TISIDB database analysis shows a negative correlation between cofilin expression and Treg abundance in COAD. C. WB analysis of cofilin, p-cofilin, ROCK2, and RhoA in Hct116 and LoVo control cells and PDCs. D. qRT-PCR analysis of cofilin mRNA levels. Statistical comparisons between groups were performed using an unpaired two-tailed Welch's t-test (*P<0.05, **P<0.01). E. IF staining showing subcellular localization of cofilin in Hct116 control cells and PDCs. F. IF staining showing subcellular localization of cofilin in LoVo control cells and PDCs. G. siRNA-mediated knockdown of cofilin followed by WB analysis of RhoA, ROCK2, cofilin, and p-cofilin protein levels (NC, negative control; MC, mock; si1#, si2#, si3#, si-cofilin plasmids). H. Wound healing assays and I. Transwell invasion and migration assays assessing the invasive and migratory abilities of PDCs with negative control knockdown (si-NC) versus cofilin-knockdown (si-cofilin #2) PDCs.

*Overexpressed cofilin regulates the RhoA pathway to promote invasion and migration of PDCs*. To investigate the role of cytoskeleton-associated proteins in PDCs, we analyzed the expression patterns of key components of the cofilin signaling pathway. Initial analysis using the GEPIA database indicated that cofilin was highly expressed in colorectal adenocarcinoma (COAD) tissues compared to normal tissues (Figure 2A). Tumor immune microenvironment analysis using TISIDB further demonstrated a negative correlation between cofilin expression and regulatory T cells (Treg) infiltration (Figure 2B). To evaluate the sustained stability of CoCl₂-induced PDCs, we measured cofilin protein expression on days 2, 3, and 7 post-withdrawals, as well as in passages 1, 2, and 4 (P1, P2, P4) cells. WB showed that cofilin expression was suppressed during the initial 2-3 days, likely because of hypoxia-driven massive cell death. However, after 7 days of recovery or by passage 1, cofilin expression returned to levels comparable to those in untreated cells and remained elevated from passages 2 to 4, indicating the relative stability of the established PDC state during early passages (Fig. S1). To assess the expression of cofilin pathway proteins, WB was performed, which demonstrated that both cofilin and ROCK2 were overexpressed in PDCs compared to control Hct116 and LoVo cells. RhoA expression was higher in LoVo PDCs than in control cells, whereas no significant change was observed in Hct116 cells, which may be partially attributed to the distinct genetic backgrounds of the two CRC cell lines (Figure 2C, Fig. S1). Quantitative real-time PCR (qRT-PCR) confirmed that cofilin mRNA levels were elevated in PDCs relative to those in control cells (Figure 2D). The subcellular localization of cofilin was visualized using IF and immunocytochemical staining. IF staining revealed that cofilin was ubiquitously distributed within Hct116 and LoVo PDCs, with greater nuclear localization than in control cells (Figure 2E-2F). Immunocytochemical staining further demonstrated that, in contrast to control cells, cofilin was predominantly localized in the cytoplasm but was also present in the nucleus (Fig. S1). To investigate the impact on downstream pathway proteins, cofilin expression was efficiently knocked down using specific siRNAs, with the reduction validated by WB (Fig. S1), thereby establishing a reliable basis for subsequent expression analysis. Reduced cofilin levels led to the decreased expression of RhoA, ROCK2, and p-cofilin, suggesting that cofilin mediated the RhoA/ROCK2 signaling pathway (Figure 2G, Fig. S1). Compared to control PDCs, those with cofilin knockdown (si 2#) in both Hct116 and LoVo cells showed markedly diminished invasion and migration capabilities, as demonstrated by wound healing, Transwell invasion, and Transwell migration assays, respectively (Figure 2H-2I, Fig. S2).

**Figure 3:**
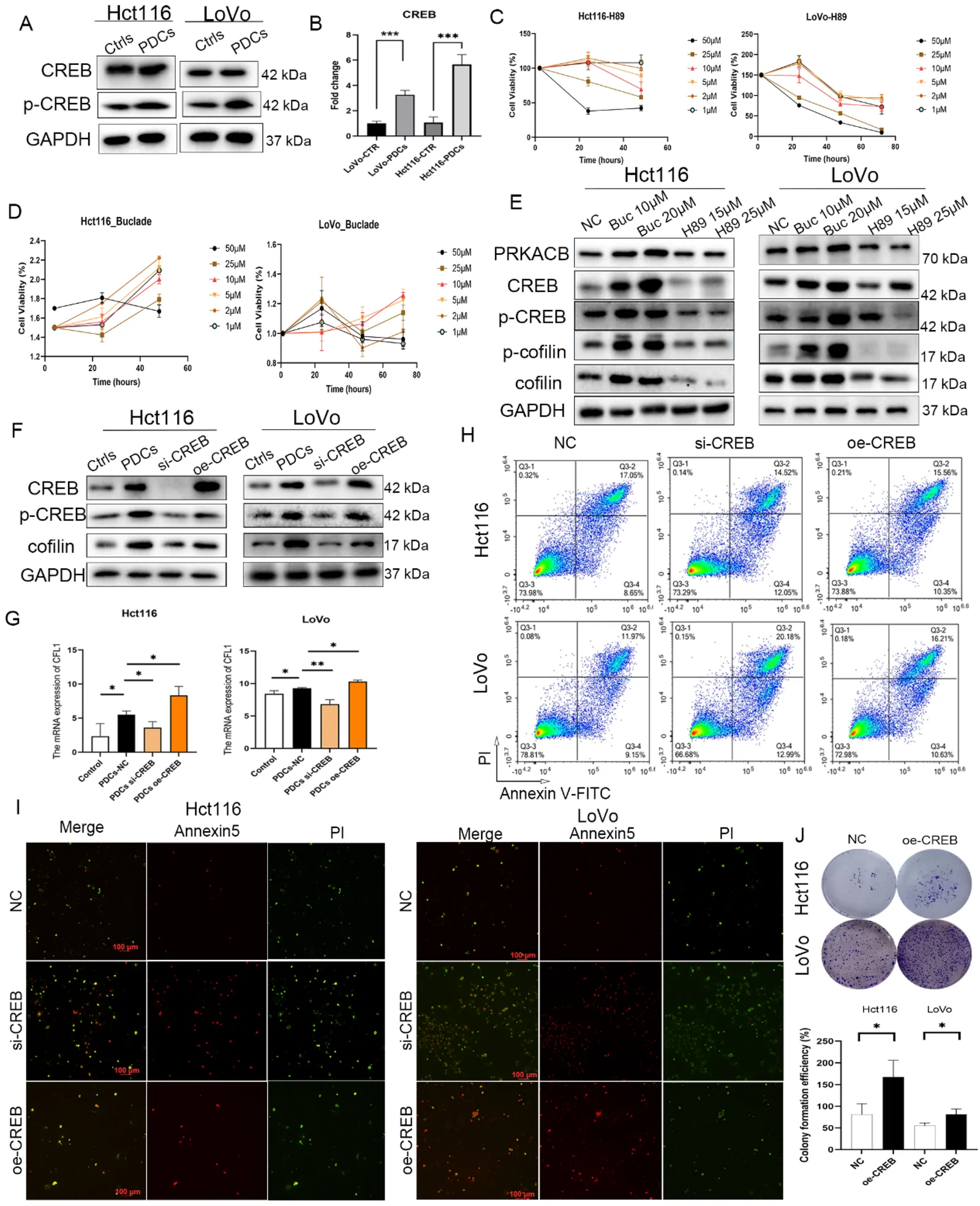
** The PKA/CREB axis regulates cofilin expression and inhibits apoptosis in PDCs.** A. WB analysis of CREB and p-CREB expression in Hct116 and LoVo control cells and PDCs. B. qRT-PCR analysis of CREB mRNA levels in Hct116 and LoVo PDCs and corresponding control cells. Comparisons between pairs of groups were performed using an unpaired two-tailed Welch's t-test. C. CCK-8 assay showing cell viability of Hct116 and LoVo PDCs following H89 treatment. D. CCK-8 assay showing cell viability of Hct116 and LoVo PDCs following Bucladesine treatment. E. WB analysis of PRKACB, CREB, p-CREB, p-cofilin, and cofilin expression in Hct116 and LoVo PDCs treated with Bucladesine or H89. F. WB analysis of CREB, p-CREB, and cofilin expression in PDCs following CREB overexpression or siRNA-mediated knockdown. G. qRT-PCR analysis of cofilin mRNA levels following CREB overexpression or knockdown in Hct116 and LoVo PDCs and their control cells. Statistical significance was determined using the Kruskal-Wallis' test followed by post-hoc analysis with Bonferroni correction. H. Annexin V/PI staining and I. Flow cytometry analysis of apoptosis following CREB knockdown or CREB overexpression. J. Colony formation assay assessing colony formation ability following CREB overexpression. Differences between two groups were determined using an unpaired two-tailed Welch's t-test. *P<0.05, **P<0.01, ***P<0.001. ov-CREB: CREB overexpression; si-CREB: CREB knockdown.

*PKA/CREB signaling inhibits PDC apoptosis and regulates cofilin expression*. Cellular signal transduction is typically initiated by first messengers that trigger intracellular receptors such as cAMP 23. Components of the cAMP signaling pathway are often upregulated in therapy-resistant cells to promote cell survival 32. As a key secondary messenger, cAMP mediates the activation of PKA and regulates cancer cell invasion and metabolism 33. First, we examined the protein levels of CREB and p-CREB, both of which are downstream effectors of the cAMP pathway. WB analysis showed that p-CREB levels were higher in PDCs than in control cells (Figure 3A and S2). Similarly, CREB mRNA levels were upregulated in PDCs (Figure 3B). To assess whether PKA, a downstream effector of cAMP, affected the cofilin pathway in PDCs, we investigated whether PKA-induced CREB phosphorylation influenced cofilin expression. Hct116 and LoVo PDCs were treated with the PKA inhibitor H89 or the PKA activator Bucladesine at gradient concentrations, and cell viability was assessed using CCK-8 assays, with absorbance measured at 24, 36, and 48 h (Figure 3C-3D). As shown in Figure 3E, treatment with Bucladesine (10 μM and 20 μM) increased the levels of PRKACB, cofilin, p-cofilin, CREB, and p-CREB. Conversely, treatment with H89 significantly decreased the p-CREB and p-cofilin levels. However, H89 affected multiple phosphorylation events independent of PKA 34. WB and qRT-PCR assays demonstrated that CREB depletion decreased both the protein and mRNA levels of cofilin, whereas CREB overexpression reversed this effect (Figure 3F-3G, Fig. S2). Furthermore, Annexin V/PI staining followed by flow cytometry demonstrated that CREB knockdown increased cell apoptosis, whereas CREB overexpression rescued cell death in both LoVo and Hct116 PDCs (Figure 3H-3I). Colony formation assays confirmed that CREB overexpression significantly increased the colony number compared to control cells (Figure 3J). Collectively, these findings demonstrate that CREB enhanced the proliferative capacity of Hct116 and LoVo PDCs.

**Figure 4:**
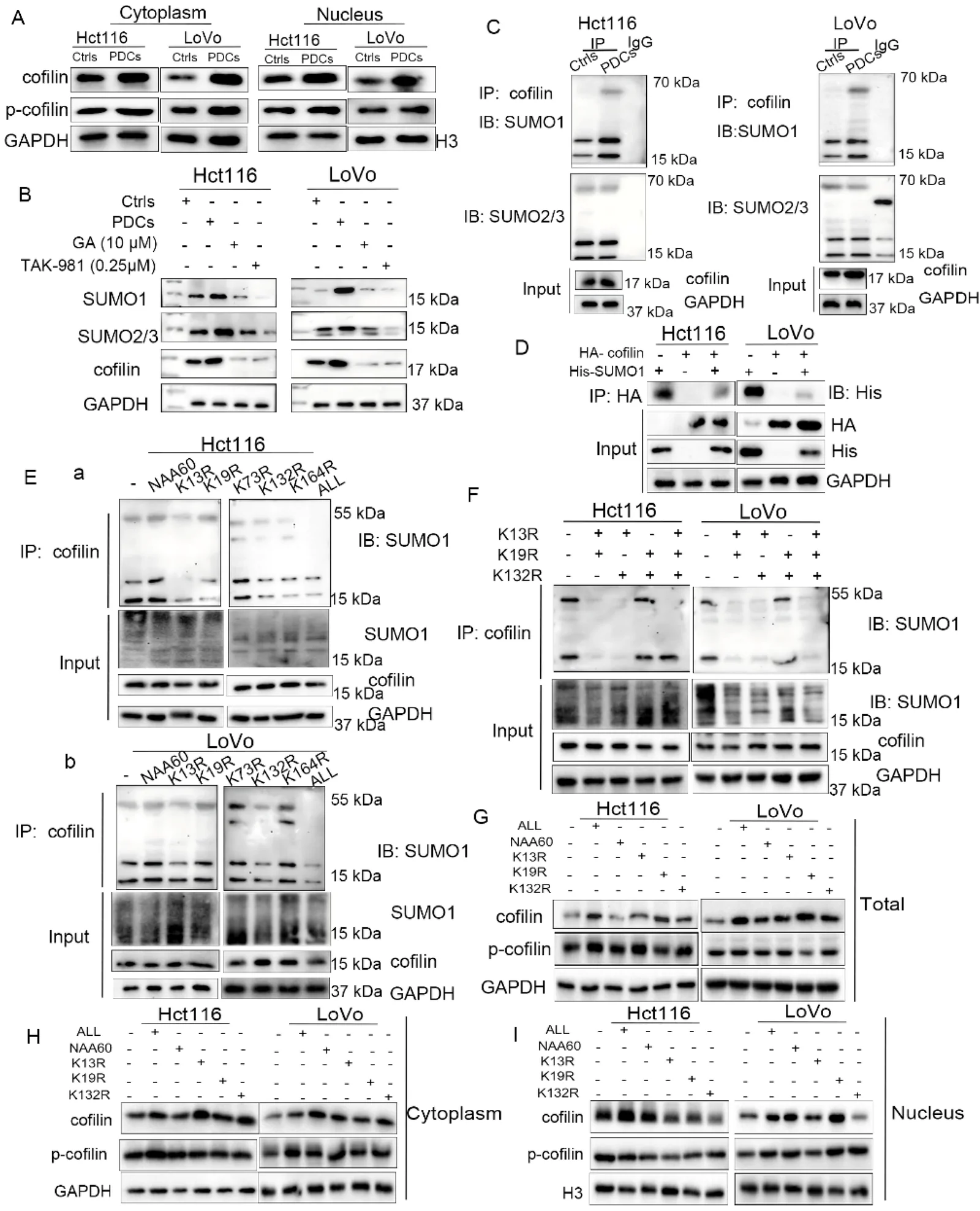
** SUMOylation of cofilin enhances its nuclear translocation.** A. Nuclear and cytoplasmic fractionation followed by WB analysis of cofilin expression in Hct116 and LoVo control cells and PDCs. B. GA and TAK-981 treatment followed by WB analysis of SUMO1, SUMO2/3 and cofilin protein levels. C. IP assay endogenous cofilin SUMOylation in Hct116 and LoVo control cells versus PDCs. D. Additional auxiliary validation using HA-tagged cofilin and His-SUMO1. E. Co-IP analysis of cofilin SUMOylation following treatment with different SUMOylation site mutants in (a) Hct116 and (b) LoVo cells. F. Co-IP detection of endogenous cofilin SUMOylation following treatment with K13R, K19R, and K132R mutants. Hct116 and LoVo PDCs were treated with K13R, K19R, K132R, ALL mutants, and NAA60; WB analysis of cofilin and p-cofilin protein levels in total (G), cytoplasmic (H), and nuclear (I) fractions.

*SUMOylation of cofilin enhances its nuclear translocation*. SUMOylation is a widespread post-translational modification that links multiple biological processes to tumor progression. We investigated whether cofilin undergoes post-translational modifications, such as SUMOylation, and how this affects its function. First, nuclear and cytoplasmic cofilin proteins were extracted, and WB analysis showed that nuclear cofilin was significantly overexpressed in Hct116 and LoVo PDCs (Figure 4A). To assess whether SUMOylation regulated cofilin levels, PDCs were treated with Subasumstat (TAK-981), a highly selective SUMOylation inhibitor. CCK-8 viability assays determined the optimal treatment conditions (0.25 µM for 24 hours) for subsequent experiments (Fig. S2). Preliminary experiments also included ginkgolic acid (GA) for comparison, although TAK-981 showed superior target specificity in the key validation experiments. WB analysis demonstrated that TAK-981 treatment reduced SUMO1, SUMO2/3, and cofilin abundance, with combined inhibitor treatment producing further suppression (Figure 4B, Fig. S2).

Furthermore, to directly verify cofilin SUMOylation, non-reducing gel electrophoresis followed by immunoblot analysis using an anti-SUMO antibody revealed that SUMO1 conjugation to cofilin occurred under endogenous physiological conditions, whereas SUMO2/3 conjugation remained unchanged in control cells and PDCs (Figure 4C). Transient transfection of HA-cofilin and His-SUMO1 recombinant plasmids into PDCs, followed by co-IP assays under exogenous conditions, confirmed that the HA tag was conjugated to the His tag (Figure 4D). These findings demonstrate that cofilin underwent SUMOylation mediated by SUMO1.

To identify potential cofilin SUMOylation site(s), we used the GPS-SUMO, SUMOplot, and JASSA databases to predict SUMOylation sites in cofilin (UniProt ID: P23528) (Table S6). By overlapping the sites predicted according to scores and ranks, we narrowed down the potential SUMOylation sites of cofilin to lysine 13 (K13), 19 (K19), 73 (K73), 132 (K132), and 164 (K164), as well as a construct with all five sites mutated (referred to as "ALL"). In addition, the N-terminal α-amino acid (NH_2_) of cofilin, previously reported as a major modification site, was examined using NAA60 to protect α-NH2 activity. Furthermore, we individually mutated each site by replacing lysine (K) with either arginine (R) to inactivate the site or glutamine (Q) to confer constitutive activity. SUMOylation assays revealed that the K13R, K132R, and K164R mutants exhibited reduced cofilin SUMOylation, whereas the NAA60 and K73R mutants had no effect (Figure 4E). Subsequently, these mutations were combined to further assess cofilin SUMOylation. Co-IP results revealed that the K13R+K19R and K13R+K132R combinations significantly reduced cofilin SUMOylation (Figure 4F). When Hct116 and LoVo PDCs were treated with the K13R, K19R, K132R, or ALL mutants, cofilin protein levels increased slightly, whereas p-cofilin expression remained unchanged (Figure 4G). Cytoplasmic and nuclear fractions analyzed by WB assays indicated that the K13R, K19R, and K132R mutants reduced cofilin translocation from the cytoplasm to the nucleus (Figure 4H-4I).

### TRIM28 mediates K132-site SUMOylation of cofilin and enhances PDC invasiveness

**Figure 5:**
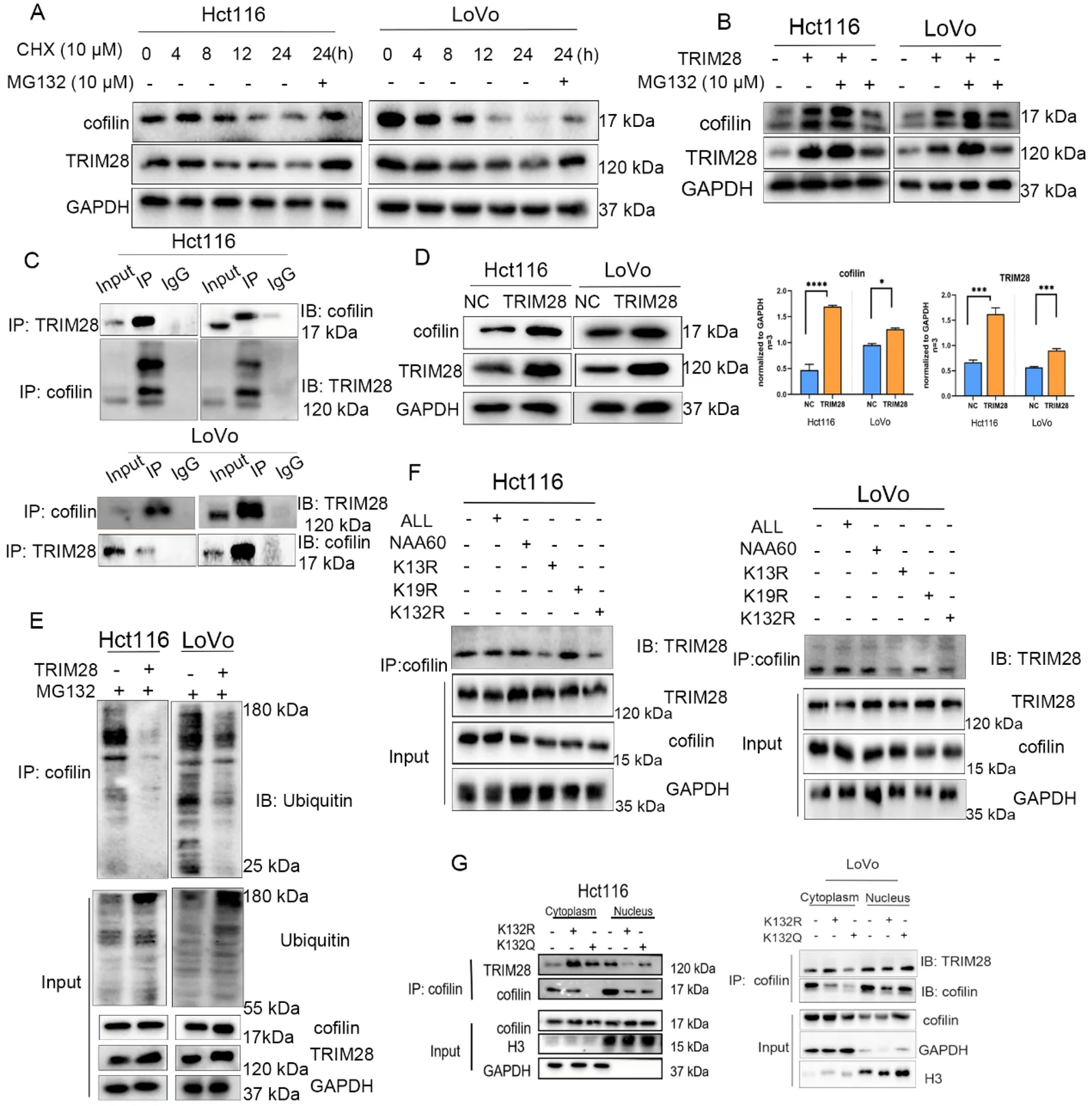
** TRIM28 mediates SUMOylation of cofilin at the K132 site and promotes its nuclear translocation.** A. PDCs were treated with CHX and MG132, and the half-lives of cofilin and TRIM28 proteins were assessed by WB. B. WB analysis of cofilin protein levels following TRIM28 and/or MG132 treatment. C. Co-IP assays showing the interaction between TRIM28 and cofilin in Hct116 and LoVo PDCs. D. WB analysis demonstrating that TRIM28 overexpression elevated cofilin protein levels. Comparisons between pairs of groups were performed using an unpaired two-tailed Welch's t-test. E. Co-IP experiments demonstrated that TRIM28 inhibited ubiquitination of cofilin. F. Co-IP experiments showing the interaction between TRIM28 and cofilin using various site-directed mutants in Hct116 and LoVo PDCs. G. Co-IP analysis of TRIM28-cofilin interaction in Hct116 and LoVo PDCs. TRIM28 interacts with cofilin in both the cytoplasm and the nucleus, as assessed using active and inactive K132-site mutants. ***P<0.001, ****P<0.0001.

TRIM28, a SUMO E3 ligase, was identified as a potential cofilin-modifying protein by database screening. To explore whether TRIM28 modulates cofilin protein stability, we examined the effects of TRIM28 on endogenous cofilin stability following treatment with the protein synthesis inhibitor cycloheximide (CHX). CHX significantly reduced the half-lives of both cofilin and TRIM28, whereas proteasome inhibition by MG132 reversed this degradation in Hct116 and LoVo PDCs (Figure 5A, Fig. S2). Moreover, cofilin protein levels were restored by MG132 in PDCs overexpressing TRIM28 (Figure 5B, Fig. S3). We investigated the relationship between TRIM28 and cofilin expression. Co-IP assays confirmed the direct interaction between TRIM28 and cofilin (Figure 5C). TRIM28 overexpression increased the protein levels of both TRIM28 and cofilin (Figure 5D, Fig. S3), whereas co-IP analysis revealed that TRIM28 substantially suppressed cofilin ubiquitination (Figure 5E), indicating that TRIM28 regulated cofilin stability by selectively inhibiting proteasomal degradation. These findings suggested that TRIM28 positively regulated cofilin expression.

Since cofilin undergoes SUMOylation, we investigated whether TRIM28 modulated this modification. The K13R and K132R mutations reduced TRIM28-cofilin binding (Figure 5F), whereas the active-site mutants, K13Q, K19Q, and K132Q, exhibited differential responses to TRIM28-mediated SUMOylation in LoVo PDCs (Fig. S3). In addition, the K132R mutant showed increased cofilin localization to the cytoplasm, whereas the K132Q mutant showed increased cofilin localization to the nucleus (Figure 5G). Furthermore, TRIM28 treatment promoted cofilin SUMOylation, which enhanced the nuclear translocation of p-CREB, whereas CREB remained localized to both the cytoplasm and nucleus (Fig. S3). Transwell invasion and migration assays demonstrated that cofilin SUMOylation-defective mutants (K13R, K19R, and K132R; designated cofilin^mut^) exhibited reduced invasion and migration compared to PDCs, while TRIM28 overexpression partially rescued these defects (Fig. S4). In summary, these findings suggest that TRIM28 inhibits cofilin ubiquitination and degradation while promoting its SUMOylation at K132, thereby facilitating cofilin nuclear translocation and enhancing the invasion and migration capacities of PDCs.

**Figure 6:**
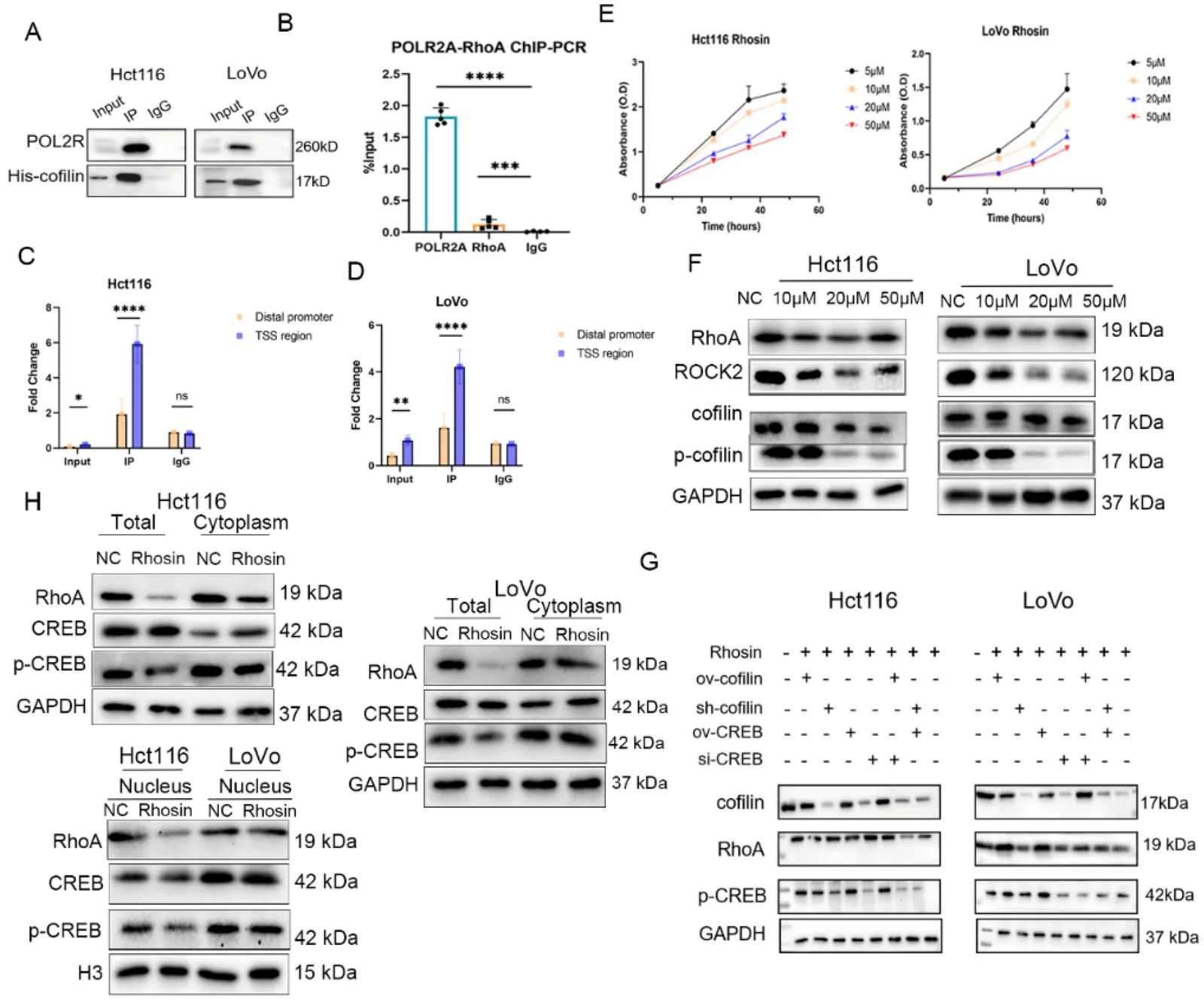
** Nuclear cofilin facilitates RNA polymerase II occupancy on *RHOA* and enhances its transcription.** A. Co-IP assays showing the interaction between POLR2A and cofilin in the nuclear fraction of Hct116 and LoVo PDCs. B. ChIP-PCR assay detecting POLR2A binding to the *RHOA* promoter. C. ChIP-PCR assay showing POLR2A interaction with the TSS and distal promoter region of *RHOA* in Hct116 PDCs. D. Same as (C) but performed in LoVo PDCs. Comparisons between pairs of groups were performed using an unpaired two-tailed Welch's t-test. E. PDCs were treated with the RhoA inhibitor Rhosin, and cell absorbance was measured by CCK-8 assay. F. Different concentrations of Rhosin were applied to assess protein levels of RhoA, ROCK2, cofilin, and p-cofilin by WB. G. WB assays detecting cofilin, RhoA, and p-CREB expression in cofilin and CREB rescue assays under Rhosin treatment conditions. H. WB analysis of total, cytoplasmic, and nuclear protein expression of RhoA, CREB, and p-CREB in Hct116 and LoVo PDCs treated with 20 μM Rhosin. ov-cofilin, cofilin overexpression; sh-cofilin, cofilin knockdown; ov-CREB, CREB overexpression; si-CREB, CREB knockdown; *P<0.05, **P<0.01, ***P<0.001, ****P<0.0001, n.s., non-significant.

*Nuclear cofilin may recruit RNA polymerase II to the RHOA promoter*. Previous studies have indicated that cofilin can be recruited to transcriptional start sites (TSS) and proximal promoter regions, where it interacts with RNA polymerase II (POLR2A) to enhance transcriptional activity. We hypothesized that nuclear cofilin would promote *RHOA* transcription. To demonstrate the cofilin-POLR2A interaction, we used His-tagged cofilin because of its low endogenous nuclear cofilin abundance. Co-IP assays verified a direct interaction between cofilin and POLR2A in Hct116 and LoVo PDCs (Figure 6A). ChIP-PCR revealed *RHOA*-POLR2A binding in the nucleus (Figure 6B), which was confirmed by ChIP-seq database analysis showing POLR2A occupancy at the *RHOA* promoter in the UCSC Genome and ENCODE databases 35, 36 (Fig. S4). To assess cofilin-dependent *RHOA* regulation, we designed primer pairs targeting the *RHOA* TSS-proximal and distal promoter regions (Table S3). ChIP-qPCR analysis demonstrated selective enrichment of cofilin at the TSS-proximal promoter (Figure 6C-6D), which substantially exceeded the enrichment at the distal promoter regions. Collectively, these targeted ChIP assays demonstrated that cofilin selectively accumulated in the *RHOA* TSS-proximal region to enhance transcription.

*RhoA inhibition decreases CREB phosphorylation and nuclear accumulation*. To investigate the role of *RHOA* in the proposed feedback loop, we used Rhosin, a selective *RHOA* inhibitor. CCK-8 assays were performed to determine the optimal Rhosin concentration (Figure 6E). WB results demonstrated that Rhosin decreased RhoA protein expression and suppressed the expression of cofilin and p-cofilin (Figure 6F). To validate the protein expression profiles within the proposed feedback loop, we modulated CREB and cofilin expression in PDCs using overexpression and knockdown strategies, followed by WB analysis. Rescue experiments demonstrated that Rhosin treatment combined with CREB or cofilin knockdown synergistically suppressed the target expression. Notably, overexpression of CREB in cofilin-overexpressing cells, or conversely, overexpression of cofilin in CREB-overexpressing cells, substantially reversed the Rhosin-induced suppression (Figure 6G, Fig. S4). Subcellular fractionation analysis further revealed that Rhosin treatment decreased both total and nuclear p-CREB levels without affecting cytoplasmic p-CREB levels, whereas total CREB expression remained unchanged (Figure 6H, Fig. S5). Collectively, these findings suggest that the signaling pathway operates via a positive-feedback mechanism.

**Figure 7:**
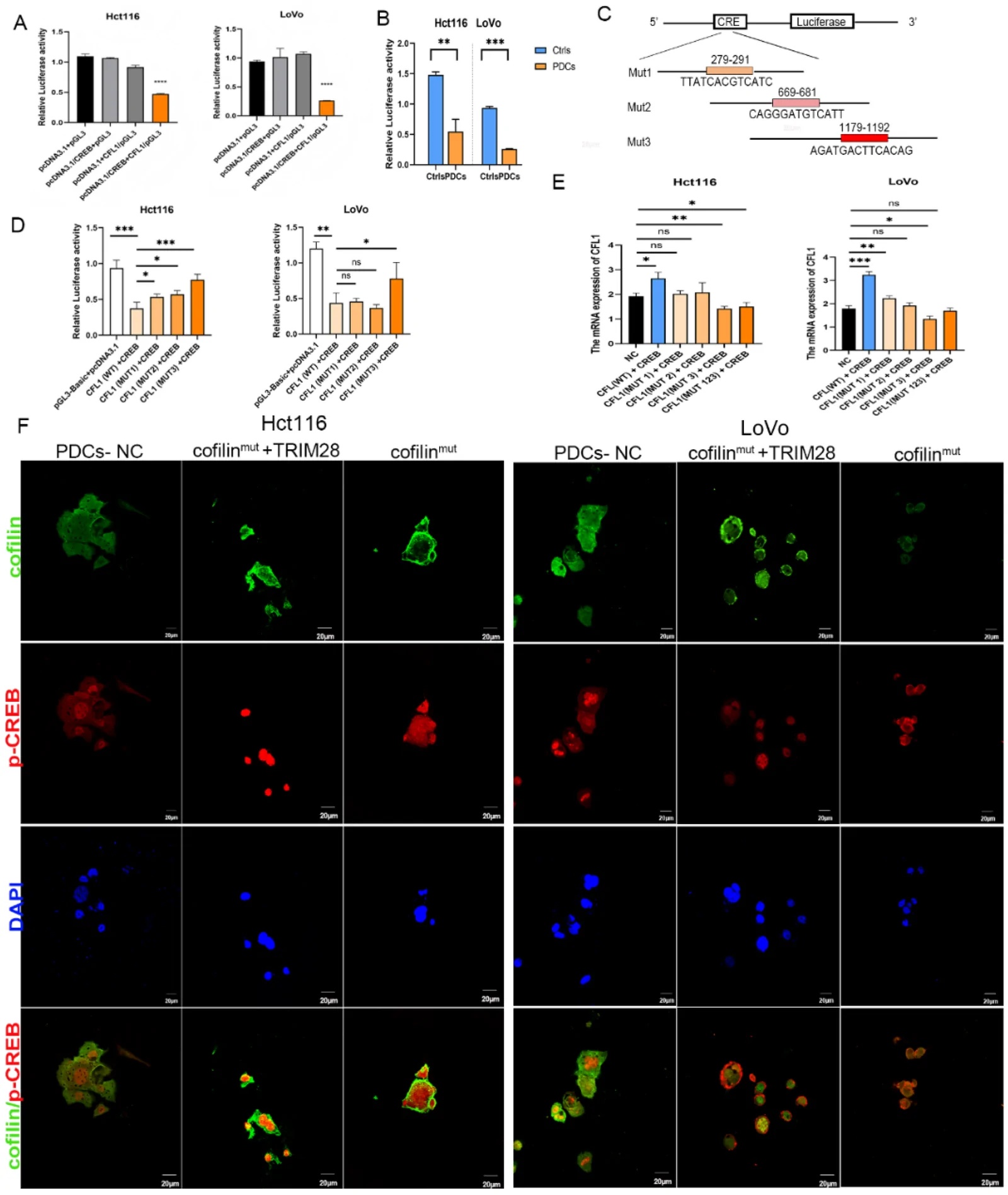
** CREB promotes cofilin transcription and expression.** A. Relative luciferase reporter assays in Hct116 and LoVo PDCs assessing interaction between *cofilin* promoter (pGL3/CFL1) and transcription factor CREB (pcDNA3.1/CREB), as well as with empty vectors. B. Luciferase activity in Hct116 and LoVo cells and PDCs following co-transfection of truncated *cofilin* promoter constructs with wild-type CREB. Comparisons between pairs of groups were performed using an unpaired two-tailed Welch's t-test. C. Schematic of three predicted binding sites within the *cofilin* promoter and construction of three corresponding mutants. D. Luciferase activity of CREB and *cofilin* mutants in Hct116 and LoVo PDCs. (WT, wild type; MUT1: mutant1; MUT2, mutant2; MUT3, mutant3). Statistical significance was determined using the Kruskal-Wallis test followed by post-hoc analysis with Bonferroni correction. E. qRT-PCR analysis of cofilin mRNA levels in Hct116 and LoVo PDCs expressing different mutants. Statistical significance was determined using the Kruskal-Wallis test followed by post-hoc analysis with Bonferroni correction. F. IF staining shows that TRIM28-mediated cofilin nuclear translocation promotes nuclear accumulation of p-CREB. cofilin^mut^: K13R, K19R and K132R mutants; *P<0.05, **P<0.01, ***P<0.001, ****P<0.0001, n.s., non-significant.

*CREB promotes cofilin transcription and expression*. Given that CREB is a transcription factor, we hypothesized that it directly promotes cofilin transcription. To test this, we performed dual-luciferase reporter assays in Hct116 and LoVo PDCs co-transfected with a *cofilin* promoter-driven firefly luciferase construct, a wild-type CREB expression plasmid, and a Renilla luciferase reporter for normalization. Firefly signals were normalized to Renilla activity, and the results were expressed as relative luciferase activities. Serial truncations of the *cofilin* promoter were transfected into Hct116 and LoVo PDCs, and reporter assays showed that CREB bound to and transactivated the native cofilin promoter (Figure 7A). Compared with control cells, co-transfection with pcDNA3.1/CREB and pGL3/CFL1 significantly reduced relative luciferase activity in both Hct116 and LoVo PDCs (Figure 7B). Bioinformatic screening using the JASPAR and TFDB databases predicted several CREB-binding sites within the *cofilin* promoter region (Fig. S5). Three *cofilin* promoter regions containing the predicted CREB sites were mutated: MUT1 (279-291), MUT2 (669-681), and MUT3 (1179-1192) (Figure 7C). In Hct116 PDCs, all three mutants exhibited increased relative luciferase activity compared with the WT-CFL1 + CREB group. However, in LoVo PDCs, only the MUT3 mutant showed a significant increase, suggesting that MUT3 represents a critical regulatory element, at least in LoVo cells (Figure 7D). These findings were further corroborated by qRT-PCR analysis of cofilin mRNA expression across the various co-transfection groups. Compared with the NC (Hct116 and LoVo PDCs separately) group, the WT-CFL1+ CREB group exhibited increased cofilin mRNA levels in both cell lines. In Hct116 cells, the MUT3 + CREB and MUT123 (triple-mutant) groups exhibited markedly reduced mRNA expression, whereas the MUT1 and MUT2 mutants had no significant effect. In LoVo cells, both WT-CFL1+ CREB and MUT1+ CREB enhanced cofilin mRNA levels, while the MUT3 + CREB group significantly downregulated its expression (Figure 7E). These results identify the MUT3 region as a critical regulatory site within the *cofilin* promoter. IF assays showed that the inactive SUMOylation mutant (cofilin^mut^) increased the cytoplasmic colocalization of cofilin with p-CREB, whereas TRIM28 treatment and active SUMO mutants promoted nuclear colocalization (Figure 7F).

**Figure 8:**
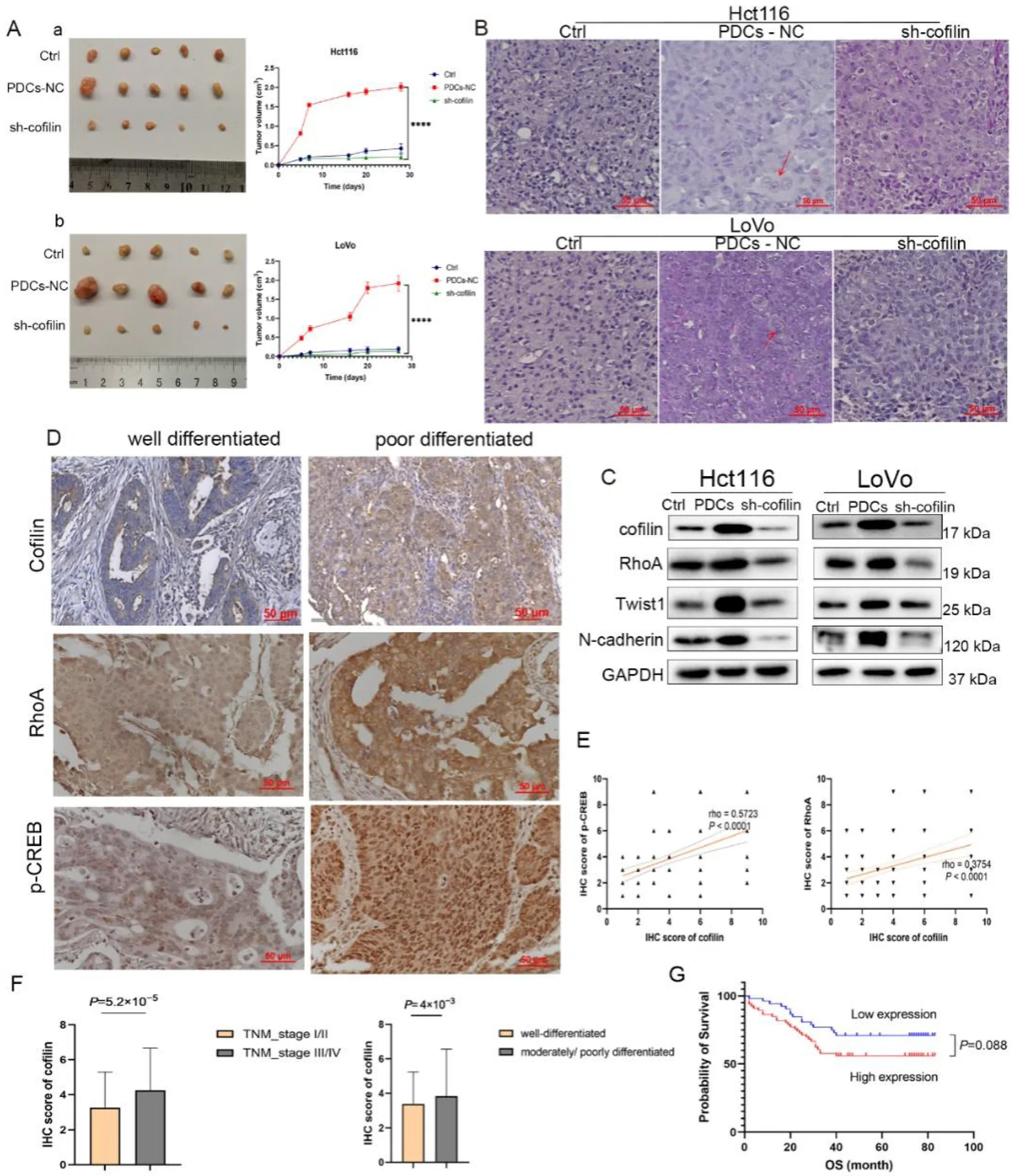
** Cofilin inhibits xenograft tumor growth from Hct116 and LoVo PDCs and its own expression in CRC tissues.** A. Xenograft tumors induced by Hct116 (a) and LoVo (b) control cells, PDC-NC, and sh-cofilin PDCs, with tumor volume curves measured over time. B. H&E staining of Hct116 and LoVo xenograft tumors. Red arrows indicate PGCCs. C. WB analysis of RhoA, cofilin, and EMT protein levels in control, PDC-NC, and sh-cofilin groups in Hct116 and LoVo xenograft tumors. D. Representative IHC staining of cofilin, RhoA, and p-CREB in well-differentiated and poorly differentiated tissues from the same tissue microarray cohort. E. IHC scores showing positive correlations between cofilin expression and p-CREB and RhoA expression, respectively. F. Correlation of cofilin IHC scores with TNM stage and histological grade in 119 CRC tissue microarray samples. G. Correlation between cofilin expression and OS in 119 CRC patients. Ctrl, control cells; PDC-NC, negative control; sh-cofilin, knockdown of cofilin in PDCs; ****P<0.0001, n.s., non-significant.

*Knockdown of cofilin inhibits PDCs-derived xenograft tumors.* To further assess the role of cofilin *in vivo*, we performed a shRNA-mediated knockdown of cofilin. Hct116 and LoVo PDCs were transduced with lentiviruses and injected subcutaneously into nude mice to establish xenograft tumors. The mice were divided into three groups: control, PDCs-NC, and sh-cofilin PDCs. Tumors in the PDCs-NC group were larger than those in the control group, whereas cofilin knockdown significantly inhibited xenograft tumor growth (Figure 8A). These findings demonstrated that cofilin knockdown reduced the tumorigenic capacity of Hct116 and LoVo PDCs *in vivo*. Hematoxylin and eosin staining revealed that xenograft tumors derived from LoVo and Hct116 PDCs contained more giant and polyploid cells than those derived from control cells, whereas sh-cofilin reduced the number of PGCCs and increased cell death (Figure 8B). IHC staining revealed stronger cofilin expression in tumors derived from Hct116 and LoVo PDCs than in control tumors, and cofilin knockdown decreased nuclear cofilin expression (Fig. S5). WB analysis indicated that cofilin, RhoA, Twist1, and N-cadherin levels were higher in Hct116 and LoVo PDCs than in control cells, whereas cofilin knockdown downregulated cofilin, RhoA, and N-cadherin expression compared to PDCs cells (Figure 8C). Collectively, these results suggest that cofilin mediates the tumorigenic capacity of PDCs *in vivo* through the RhoA and EMT pathways.

*Clinical relevance.* A tissue microarray containing 119 CRC specimens was constructed, including 77 well-differentiated and 42 moderately_to_poorly differentiated tissue samples. The clinicopathological characteristics were summarized in Table S5. IHC was performed to evaluate the expression of cofilin, RhoA, and p-CREB in the same CRC patients' cohort (Figure 8D). Consistent with cofilin expression, RhoA and p-CREB levels were significantly elevated in cancer tissues and positively correlated with cofilin expression (Figure 8E), further supporting the pathological relevance of the cofilin/RhoA/p-CREB regulatory loop. Logistic regression analysis revealed that high cofilin expression was significantly correlated with TNM stage III/IV *(P*= 5.2×10^-5^) and moderate-to-poor differentiation (*P*= 4×10^-3^) (Figure 8F). Cox regression analysis identified TNM stage *(P*= 9×10^-6^) as the only independent prognostic factor for overall survival in patients with CRC, whereas cofilin expression (*P*= 0.088) was not associated with survival outcomes (Figure 8G, Table S5). Notably, the TCGA database and Kaplan-Meier plotter analyses indicated that lower cofilin expression was associated with poor overall survival in COAD (Fig. S5), warranting further investigation into the potential context-dependent prognostic significance.

## Discussion

PGCCs are tumor cells characterized by multiple nuclei or a single enlarged nucleus, typically arising from cell fusion or abnormal cell cycle progression. Recent studies have demonstrated that PGCCs can be induced in cancer cells resistant to chemotherapeutic agents, such as paclitaxel 37 and cisplatin 38. Moreover, the prevalence of PGCCs is elevated in high-grade malignancies and is correlated with accelerated recurrence and poor patient survival 39. Our previous study confirmed that PGCCs undergo dedifferentiation and generate highly invasive daughter cells (PDCs) through asymmetric division, thereby promoting tumor recurrence and metastasis. These PDCs undergo EMT, during which epithelial cells with cobblestone-like morphology progressively convert into spindle-shaped mesenchymal cells. This transition is accompanied by cytoskeletal alterations, downregulation of cell-cell junction proteins, and upregulation of mesenchymal markers. Previous studies have reported that dysregulated activation of the RhoA-ROCK1 pathway modulates the cytoskeletal network, leading to increased cellular stiffness and enhanced migratory persistence in PGCCs 40. Cofilin, an ADF, is frequently overexpressed in various cancer types and promotes metastasis by regulating cytoskeletal dynamics, lamellipodium formation, and EMT. Emerging evidence suggests that cofilin expression influences the therapeutic sensitivity and resistance to therapy in tumor cells. In the present study, we demonstrated that cofilin was significantly upregulated in CRC-derived PDCs, which conferred enhanced invasive and migratory capabilities. Therefore, understanding the molecular mechanisms governing cofilin expression and stability in these cells is critical.

Beyond its classical cytoplasmic functions, we observed that cofilin contributed to the invasive potential of Hct116 and LoVo PDCs through its nuclear localization. Previous reports have indicated that while p-cofilin restricts protrusion shape by regulating cortical actin, cofilin itself can translocate to the nucleus to maintain structural integrity and genomic function 41-43. This translocation is facilitated by a nuclear localization signal that enables cofilin to transport actin via the importin pathway and potentially mediate the nuclear entry of signaling molecules like β-catenin and NF-κB 44-47. Concurrently, we found that SUMOylation, a post-translational modification essential for nuclear transport, cell cycle progression, protein homeostasis, and the maintenance of cancer stem cell self-renewal 48, was markedly upregulated in PDCs. Specifically, co-IP assays confirmed that cofilin undergoes SUMOylation at residues K13, K19, and K132, which collectively govern its cytoplasmic-to-nuclear translocation. To ensure mechanistic rigor, these findings were validated by endogenous immunoprecipitation, with site-directed mutagenesis and exogenous SUMO overexpression serving as auxiliary tools for site mapping. Nevertheless, future high-bandwidth analyses, such as mass spectrometry, are required to precisely characterize the SUMOylation dynamics.

TRIM28, also known as Krüppel-associated box-associated protein 1 or transcriptional intermediary factor 1β, plays complex roles in tumorigenesis and cancer progression. Evidence indicates that the phosphorylated form of TRIM28 regulates POLR2A and other transcription factors that drive gene expression 49, 50. Depending on its structure, TRIM28 functions as an E3 ligase that mediates SUMOylation and ubiquitination 51. In addition to inhibiting the ubiquitination of pro-tumor proteins, TRIM28 mediates SUMOylation of oncogenic proteins, including PD-L1, TRIM24, and NLRP3 52-54. Our findings demonstrate that TRIM28 inhibits the ubiquitin-mediated degradation of cofilin, promotes SUMOylation of cofilin at the K132 site, enhances cofilin nuclear localization, and subsequently increases the invasion and migration capabilities of Hct116 and LoVo PDCs.

Emerging evidence suggests that nuclear cofilin enhances RNA transcription and accelerates POLR2A-mediated transcriptional elongation in response to soft substrates 55. We found that nuclear cofilin promoted the transcription of *RHOA*, a small GTPase associated with poor prognosis, microtubule instability, and tumor-related processes in CRC 56, 57. RhoA drives p-CREB activation, whereas inhibition of RhoA attenuates its nuclear accumulation. Considering that CREB is an established driver of malignancy, chemoresistance, and poor prognosis 58, its direct binding to the cofilin promoter establishes a feedforward regulatory circuit. Previous studies reported that ATF3, another member of the CREB family, is regulated by RhoA downstream effector kinase ROCK1. ROCK1 degradation leads to the accumulation of non-phosphorylated ATF3 in the nucleus and induces apoptosis 59. Although we observed some heterogeneity in total RhoA levels between LoVo and Hct116 cells, likely due to their distinct genetic backgrounds and basal signaling states, cofilin and CREB are synergistically activated in both models. This suggests that the cofilin/RhoA/CREB regulatory axis represents a conserved core mechanism driving PDC invasion and may offer a novel therapeutic target for CRC.

This study has several limitations. First, we focused primarily on CoCl₂-induced PDC formation under hypoxic conditions. Given the inherent differences in hypoxic responses, cytoskeletal dynamics, and signaling networks between normal intestinal epithelial cells and cancer cells, further verification in non-cancerous cells is required to strengthen the current conclusions. Secondly, although cofilin expression was associated with high malignancy, we did not observe a statistically significant correlation with overall survival in our cohort, thereby suggesting the need for larger clinical datasets. Finally, further investigations should prioritize *in vivo* functional and therapeutic evaluations, including combined cofilin suppression and chemotherapeutic strategies, to establish the translational value of targeting this pathway.

## Supplementary Material

Supplementary figures and tables.

## Data Availability

The data generated in this study are not publicly available.

## Abbreviations

PGCCs: polyploid giant cancer cells
PDCs: PGCCs with daughter cells
CRC: colorectal cancer
EMT: epithelial-mesenchymal transition
PKA: protein kinase A
CoCl_2_: cobalt chloride
WB: western blotting
Co-IP: Co-immunoprecipitation
qRT-PCR: quantitative real-time PCR
ChIP: chromatin immunoprecipitation
IF: immunofluorescence
IHC: immunohistochemistry
SUMOylation: small ubiquitin-like modification
CREB: cAMP response element-binding protein
Treg: regulatory T cell
CHX: cycloheximide
POLR2A: RNA polymerase II
TSS: transcriptional start site
