# Supplementary material for: Cofilin/RhoA and CREB synergistically promote invasion and migration of polyploid giant cancer cells in colorectal cancer
Source: J Cancer. 2026 Sep 3;17(9):1583–99. doi: 10.7150/jca.138960 (PMC13573303; doi:10.7150/jca.138960)
Supplement: Supplementary file 1 [file jcav17p1583s1.pdf]

Supplementary materials

Supplementary tables

Table S1. Antibodies and reagents used for Western blotting, coimmunoprecipitation and immunofluorescence.

Antibody / reagent	Manufacturer	Country	dilution
Western blotting			
cofilin	Santa Cruz	USA	1:500
p-cofilin	Santa Cruz	USA	1:500
Anti-Ubiquitin	Abcam	USA	1:500
SUMO1	Abcam	USA	1:500
SUMO2/3	Abcam	USA	1:500
CREB	Cell Signalling Technology	USA	1:1000
p-CREB (ser133)	Cell Signalling Technology	USA	1:1000
TRIM28	Cell Signalling Technology	USA	1:1000
PRKACB	Proteintech	China	1:500
RhoA	Proteintech	China	1:500
ROCK2	Proteintech	China	1:500
GAPDH	Proteintech	China	1:5000
Twist1	Proteintech	China	1:500
Snail	Proteintech	China	1:500
RPB1	CST	USA	1:500
Coimmunoprecipitation			
cofilin	Santa Cruz	USA	1:30
TRIM28	Proteintech	China	1:30
p-CREB (ser133)	Cell Signalling Technology	USA	1:50
HA	Affinity	China	1:30
FLAG	Proteintech	China	1:30
Immunofluorescence			
cofilin	Santa Cruz	USA	1:100

p-CREB	Cell Signalling Technology	USA	1:500
Alexa fluor 488/594	Invitrogen	USA	
Phalloidin	YEASEN	CN	1:200
Reagent			
RIPA buffer	Solarbio, R0010		
nuclear and cytoplasmic protein extraction	Abbkine, KTP3001		
IP buffer	Beyotime, P0013		
protein A/G agarose	ThermoFisher, 20421		
4×Hifair® III SuperMix	YEASEN, 11137		
puromycin	MCE, HY-B1743		
SYBR Green kit	YEASEN, 11143ES		
DAPI	Solarbio, C0065		
propidium iodide (PI)	Beyotime, C1052		
Annexin A5-FITC	YEASEN, 40302ES		
ChIP Kit	Absin, abs50034		
IHC kit	ZSGB-BIO, PV9000		

Table S2. Sequences of siRNAs interference.

Gene	Forward primer	Reverse primer
si-cofilin #1	GAAGGUGCGUAAGUCUUCATT	UGAAGACUUACGCACCUUCTT
si-cofilin #2	CCUCUAUGAUGCAACCUAUTT	AUAGGUUGCAUCAUAGAGGTT
si-cofilin #3	GGAUCAAGCAUGAAUUGCATT	UGCAAUUCAUGCUUGAUCCTT
si-CREB #1	ACAGCACCCACUAGCACUAUUTT	AAUAGUGCUAGUGGGUGCUGUTT
si-CREB #2	GCAGCUCAUGCAACAUCAUTT	AUGAUGUUGCAUGAGCUGCTG
si-CREB #3	GCCUGCAAACAUUAACCAUTT	AUGGUUAAUGUUUGCAGGCCC
si-SUMO1 #1	GACAGGGUGUUCCAAUGAATT	UUCAUUGGAACACCCUGUCTT
si-SUMO1 #2	GAGAAUUGCUGAUAAUCAUTT	AUGAUUAUCAGCAAUUCUCTT

si-SUMO1 #3	GGCUUGUGGUGAUAAAUAATT	UUAUUUAUCACCACAAGCCTT
Negative control	UUCUCCGAACGUGUCACGUTT	ACGUGACACGUUCGGAGAATT
si-GAPDH	UGACCUCAACUACAUGGUUTT	AACCAUGUAGUUGAGGUCATT
si- ß-actin	UGAAGAUCAAGAUCAUUGCTT	GCAAUGAUCUUGAUCUUCATT

Table S3. Nucleotide sequences of the primers

**Gene**	**Forward primer**	**Reverse primer**
CFL1	CTCGTCTTCTGCGGCTCTCG	TTCTTCACCTCCTCTGGCGTTG
CREB1	AGCCGAGAACCAGCAGAGTG	AGAGTTACGGTGGGAGCAGATG
POLR2A	TACCCAGAGACGACTGAGGG	CACGTGAAACACAGGCTTGG
GAPDH	GCACCGTCAAGGCTGAGAAC	TGGTGAAGACGCCAGTGGA
RHOA (promoter)	AGGTGTATGTGCCCACAGTG	TGTGTCCCACAAAGCCAACT
RHOA (TSS)	CCTCAGGCAACGAATCCGAG	GTCCACGGTCTGGTCTTCAG
RHOA (distance promoter)	AGGTAAGACTGTGATGCTGGAG	ATGAAGTAGGCATTTATAAAGGCA

Table S4. Inhibitors.

**Items**	**Manufacturer**	**Function**
Ginkgolic acid (GA)	MedChemExpress (MCE)	Inhibit SUMOylation
TAK-981 (Subasumstat)	MedChemExpress (MCE)	Selective inhibitor of SUMOylation
MG132	MedChemExpress (MCE)	A potent proteasome and calpain inhibitor
Cycloheximide (CHX)	MedChemExpress (MCE)	Inhibitor of protein synthesis
Bucladesine	MedChemExpress (MCE)	Activate cAMP/PKA signaling pathway
H89	MedChemExpress (MCE)	A selective inhibitor of cAMP dependent PKA

Table S5. Logistic regression analysis and Univariable cox regression analysis of clinical characteristics in 119 CRC patients.

	**Cofilin expression [1]**

Variables (N)	*P*-value	OR value	Lower 95%CI	Upper 95%CI
Age				
< 65 (57)	0.090	0.531	0.255	1.104
≥65 (62)				
Gender				
Female (58)	0.243	1.542	0.745	3.190
Male (61)				
Differentiation				
Well (77)	**0.004***	0.296	0.130	0.672
Moderate/Poor (42)				
TNM_stage				
I/II (47)	**0.000052***	0.193	0.087	0.428
III/IV (72)				
Cofilin expression [2]				
Low (53)	-	-	-	
High (66)				

	Univariable Cox Analysis		Multivariable Cox Analysis [2]	
Variables (N)	HR (95% CI)	*P*-value	HR (95% CI)	*P*-value
Age				
< 65 (57)	1.004 (0.556-1.813)	0.991	0.851 (0.455-1.592)	0.614
≥65 (62)				
Gender				
Female (58)	1.269 (0.698-2.305)	0.434	0.952 (0.502-1.805)	0.881
Male (61)				
Differentiation				
Well (77)	0.794 (0.435-1.449)	0.453	1.717 (0.903-3.265)	0.099
Moderate/Poor (42)				
TNM_stage				

I/II (47)	0.05 (0.012-0.205)	**0.000034***	0.037 (0.009-0.159)	**0.000009***
III/IV (72)				
Cofilin expression [3]				
Low (53)	0.581 (0.311-1.084)	0.088	1.144 (0.596-2.199)	0.685
High (66)				

OR odds ratio, HR hazard ratio, CI confidence interval

[1] Binary Logistic regression

[2]Cox regression model (method = Enter)

[3]Low, IHC score ≤3; High, IHC score>3

**P*<0.05 significantly

Table S6. Prediction of SUMOylation sites of cofilin (Uniprot ID: P23528)

SUMOplot Analysis Program

No.	Pos.	Group	Score
1	K132	KKLTG IKHE LQANC	0.94
2	K13	VSDGV IKVF NDMKV	0.59
3	K96	ETKES KKED LVFIF	0.48
4	K152	RCTLA EKLG GSAVI	0.33

GPS_SUMO 2.0

	Pos.	Peptide	Score
Exp.	73	DPYATFVKMLPDKDC	0.8873
Exp.	13	AVSDGVIKVFNDMKV	0.8871
Exp.	132	KKKLTGIKHELQANC	0.8443
Exp.	53	NIILEEGKEILVGDV	0.8415
Pred.	95	TYETKESKKEDLVFI	0.8329

JASSA

Pos.	Sequece	Score
K132	DAIKKKLTGIKHELQANCYEE	High
K19	DGVIKVFNDMKVRKSSTPEEV	High

K164	GGSAVISLEGKPL--------	High
K30	VRKSSTPEEVKKRKKAVLFCL	Low
K44	KAVLFCLSEDKKNIILEEGKE	Low
K95	YDATYETKESKKEDLVFIFWA	Low

Supplementary figures and figure legends

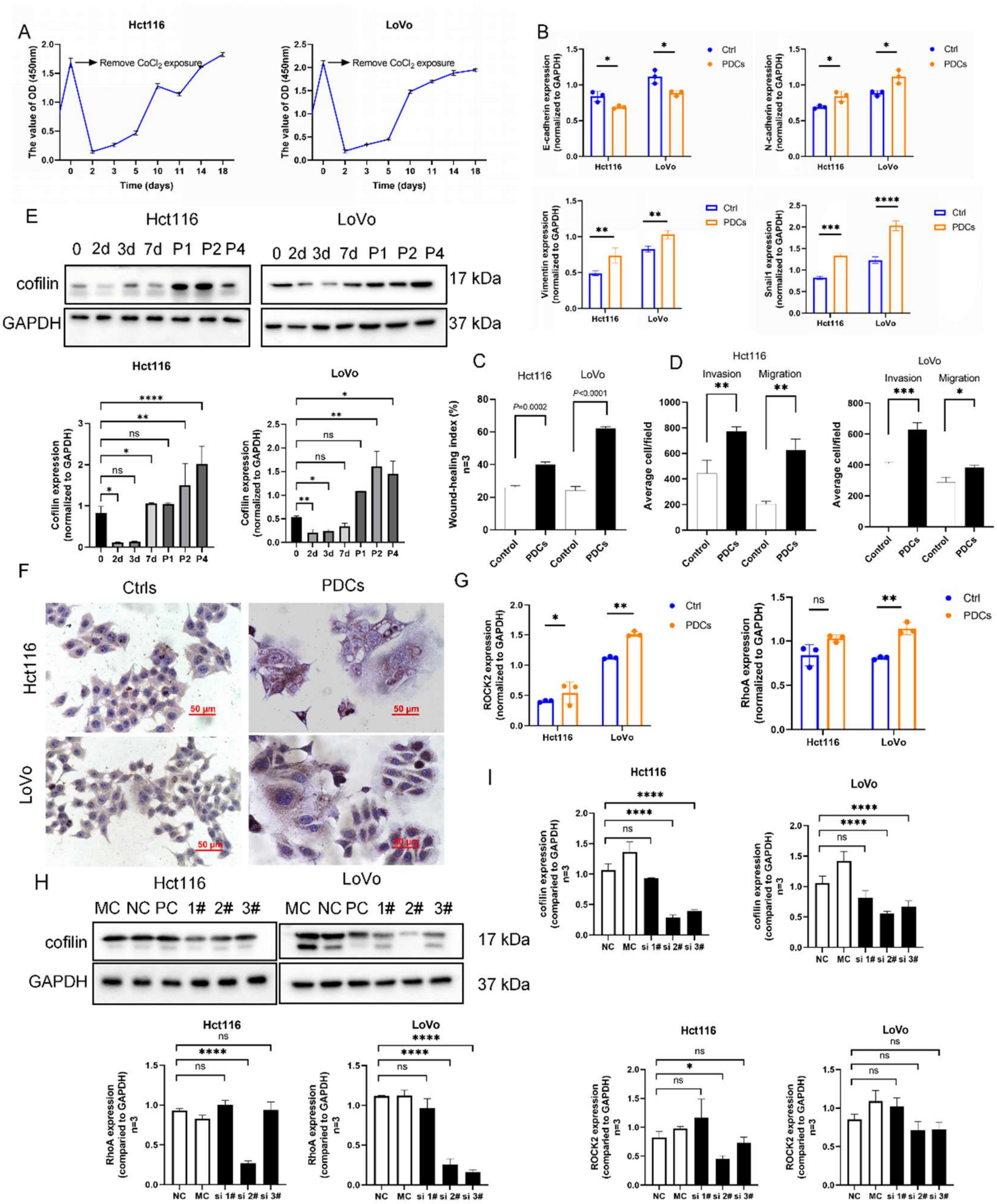

Fig. S1. A. Cell viability was assessed using a CCK-8 assay following $CoCl_2$ withdrawal. B. Statistical analysis of WB results showing the expression of EMT-related proteins in Hct116 and LoVo control cells and PDCs. Comparisons between pairs of groups were performed using an unpaired two-tailed Welch's t-test. C. Statistical analysis of wound healing assays in control cells and PDCs. D. Statistical analysis of Transwell invasion and migration assays in control cells and PDCs. Comparisons between pairs of groups were performed using an unpaired two-tailed Welch's t-test. E. WB analysis of cofilin protein expression at days 2, 3, and 7 post-withdrawals, as well as in cells at passages 1, 2, and 4 (P1, P2, P4). Statistical

significance was determined using the Kruskal-Wallis' test followed by post-hoc analysis with Bonferroni correction. F. Immunocytochemical staining of cofilin in Hct116 and LoVo control cells and PDCs. Scale bar = 50 μm. G. Statistical analysis of WB results showing the expression levels of RhoA and ROCK2 proteins. Comparisons between pairs of groups were performed using an unpaired two-tailed Welch's t-test. H. WB validation of three small interfering RNA (siRNA) plasmids used to knock down cofilin expression. Statistical significance was determined using the Kruskal-Wallis' test followed by post-hoc analysis with Bonferroni correction. I. Statistical analysis of WB results showing the expression levels of RhoA/ROCK2/cofilin pathway proteins after transfection with si-cofilin plasmids. Comparisons between pairs of groups were performed using an unpaired two-tailed Welch's t-test.

NC, negative control; MC, mock; PC, positive control; si1#, si 2#, si 3#, si-cofilin plasmids; *P<0.05, **P<0.01, ***P<0.001, ****P<0.0001, n.s., non-significant.

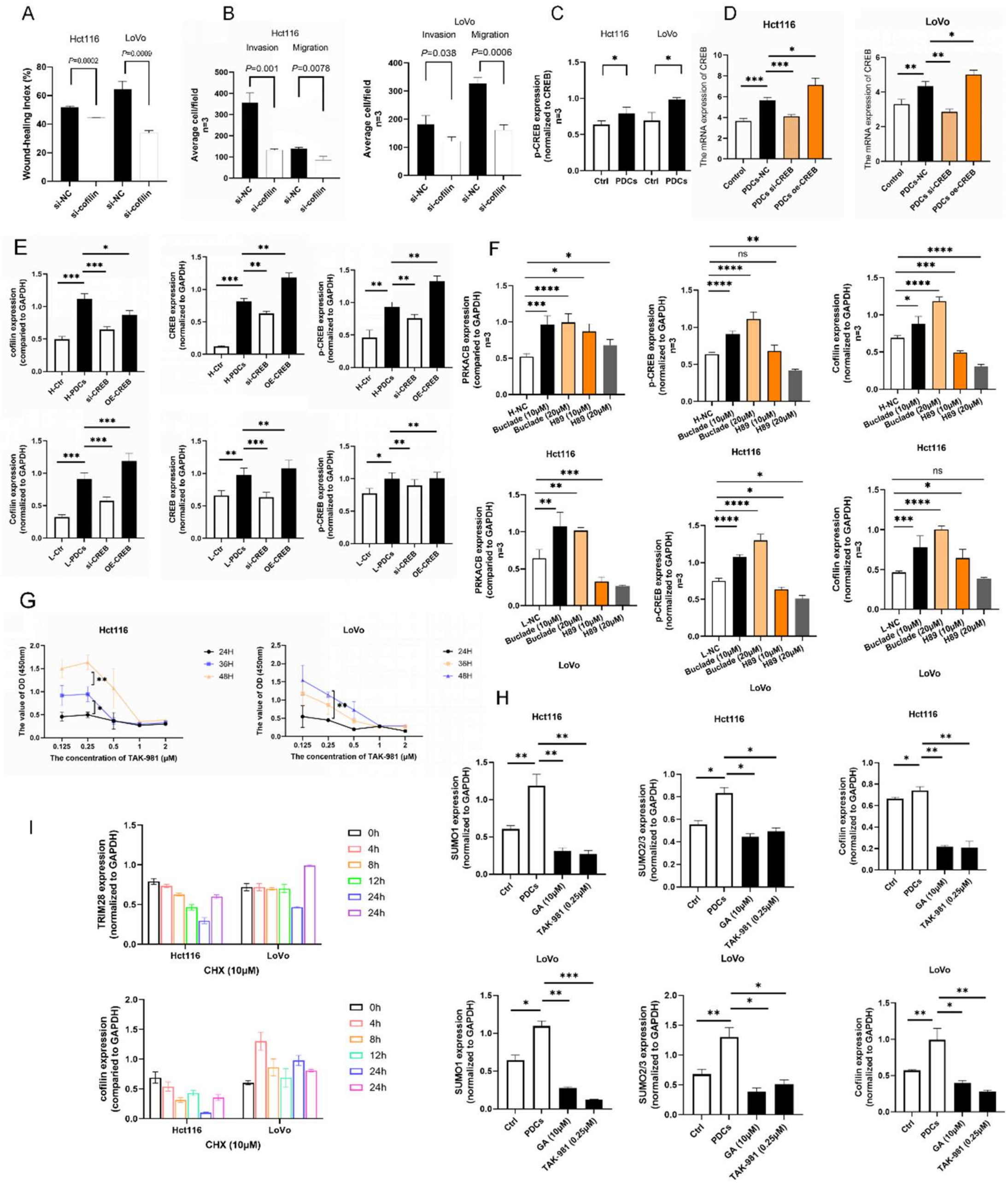

Fig. S2. A. Statistical analysis of wound healing assays in Hct116 and LoVo cells transfected with si-NC or si-cofilin. Comparisons between pairs of groups were performed using an unpaired two-tailed Welch's t-test. B. Statistical analysis of Transwell invasion and migration assays in Hct116 and LoVo cells transfected with si-NC or si-cofilin. Comparisons between pairs of groups were performed using an unpaired two-tailed Welch's t-test. C. Statistical analysis of WB results showing p-CREB expression in Hct116 and LoVo control cells and PDCs. Comparisons between pairs of groups were performed using an unpaired two-tailed Welch's t-test. D. Statistical analysis of qRT-PCR results showing mRNA levels of CREB and cofilin in

PDCs following overexpression or siRNA-mediated knockdown of CREB. Statistical significance was determined using the Kruskal-Wallis' test followed by post-hoc analysis with Bonferroni correction. E. Statistical analysis of WB showing CREB and cofilin expression after CREB overexpression or knockdown. Statistical significance was determined using the Kruskal-Wallis' test followed by post-hoc analysis with Bonferroni correction. F. Statistical analysis of WB showing PRKACB, CREB, p-CREB, cofilin, and p-cofilin expression in Hct116 and LoVo PDCs treated with Bucladesine or H89. Statistical significance was determined using the Kruskal-Wallis' test followed by post-hoc analysis with Bonferroni correction. G. CCK-8 assays showing cell viability after treatment with TAK-981 in Hct116 and LoVo PDCs. H. Statistical analysis of WB results showing cofilin protein levels after treatment with GA and/or TAK-981. Statistical significance was determined using the Kruskal-Wallis' test followed by post-hoc analysis with Bonferroni correction. I. Statistical analysis of WB results showing cofilin and TRIM28 protein levels after treatment with CHX and MG132.

si-NC, negative control cell knockdown; si-cofilin, cofilin knockdown; *$P<0.05$, **$P<0.01$, ***$P<0.001$, ****$P<0.0001$.

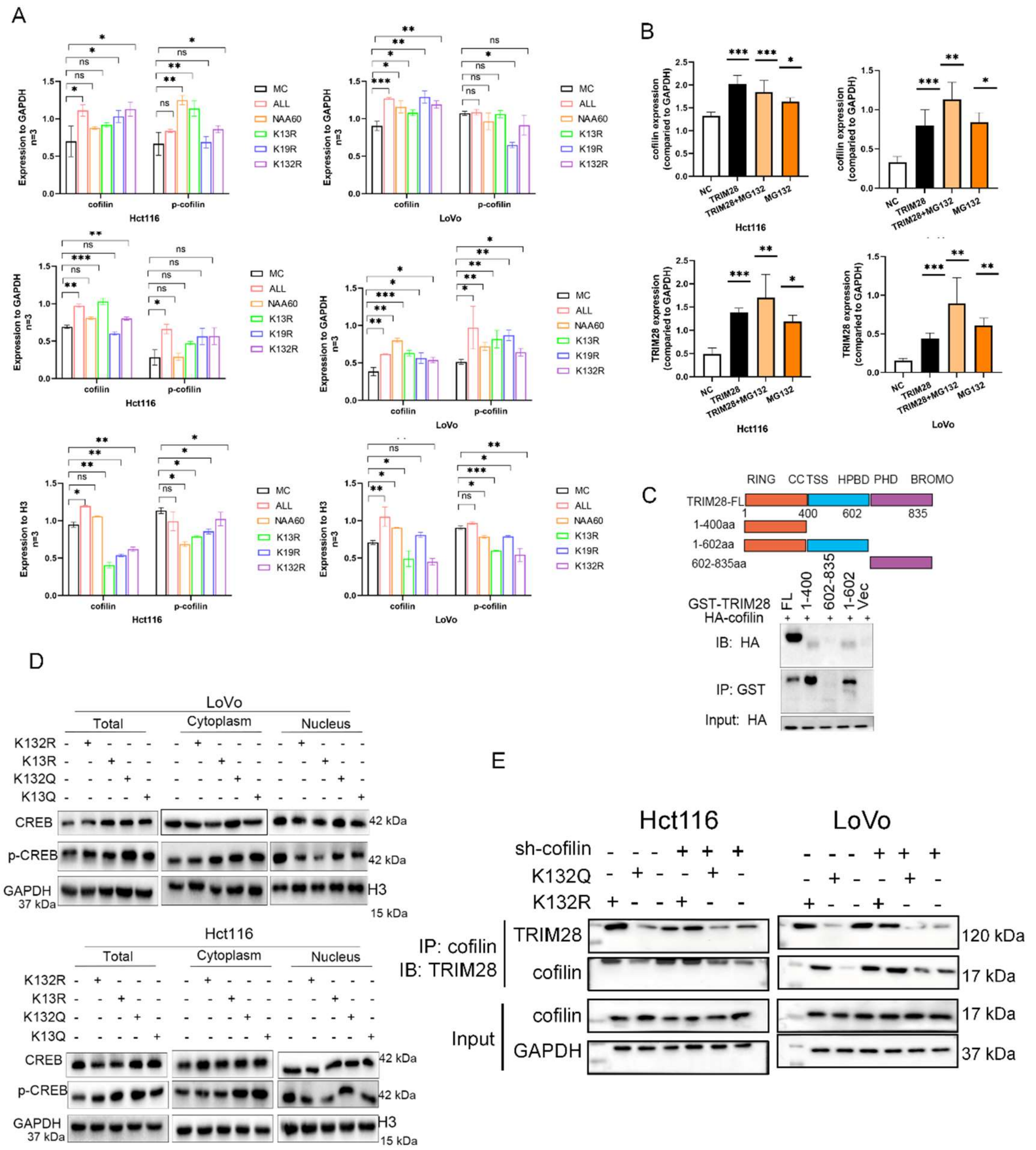

Fig. S3 A. Statistical analysis of WB results showing total, cytoplasmic, and nuclear protein levels of cofilin and p-cofilin following treatment with K13R, K19R, K132R, ALL mutants, and NAA60. Statistical significance was determined using the Kruskal-Wallis' test followed by post-hoc analysis with Bonferroni correction. B. Statistical analysis of WB showing cofilin protein levels after TRIM28 and MG132 treatment. Statistical significance was determined using the Kruskal-Wallis; test followed by post-hoc analysis with Bonferroni correction. C. WB analysis of total, cytoplasmic, and nuclear CREB and p-CREB expression in Hct16 and LoVo PDCs transfected with K132 and K13 active (Q) or inactive (R) mutants. D. Co-IP experiments assessing the

interaction of TRIM28 and cofilin in K132 active and inactive mutants under cofilin knockdown conditions; *$P<0.05$, **$P<0.01$, ***$P<0.001$, ****$P<0.0001$, n.s., non-significant.

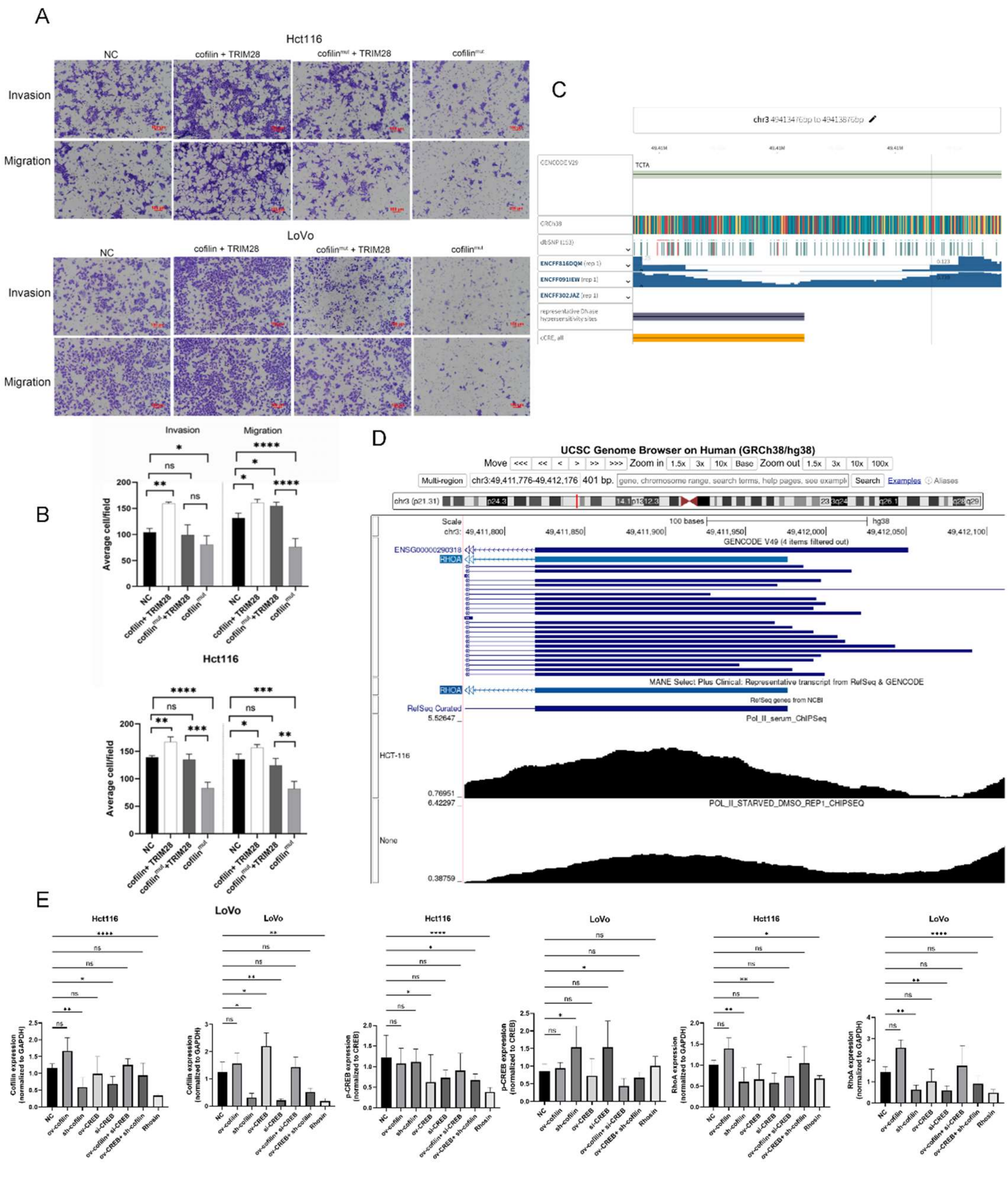

Fig. S4. A. Transwell invasion and migration assays in Hct116 and LoVo PDCs treated with cofilin+TRIM28, cofilin^{mut}+TRIM28, or cofilin^{mut} alone. Cofilin^{mut}: cofilin mutants (K13R, K19R, and K132R). B. Statistical analysis of Transwell invasion and migration assays in Hct116 and LoVo PDCs treated as described in (A). Statistical significance was determined using the Kruskal-Wallis' test followed by post-hoc analysis with Bonferroni correction. C. ChIP-seq profiles showing POLR2A occupancy

at the *RhoA* promoter in the UCSC Genome Browser. D. ChIP-seq profiles showing POLR2A occupancy at the RhoA promoter in the ENCODE database. E. Statistical analysis of WB results showing cofilin, RhoA, and p-CREB expression in cofilin and CREB rescue assays. Statistical significance was determined using the Kruskal-Wallis' test followed by post-hoc analysis with Bonferroni correction.

ov-cofilin, cofilin overexpression; sh-cofilin, cofilin knockdown; ov-CREB, CREB overexpression; si-CREB, CREB knockdown; *P<0.05, **P<0.01, ***P<0.001, ****P<0.0001, n.s., non-significant.

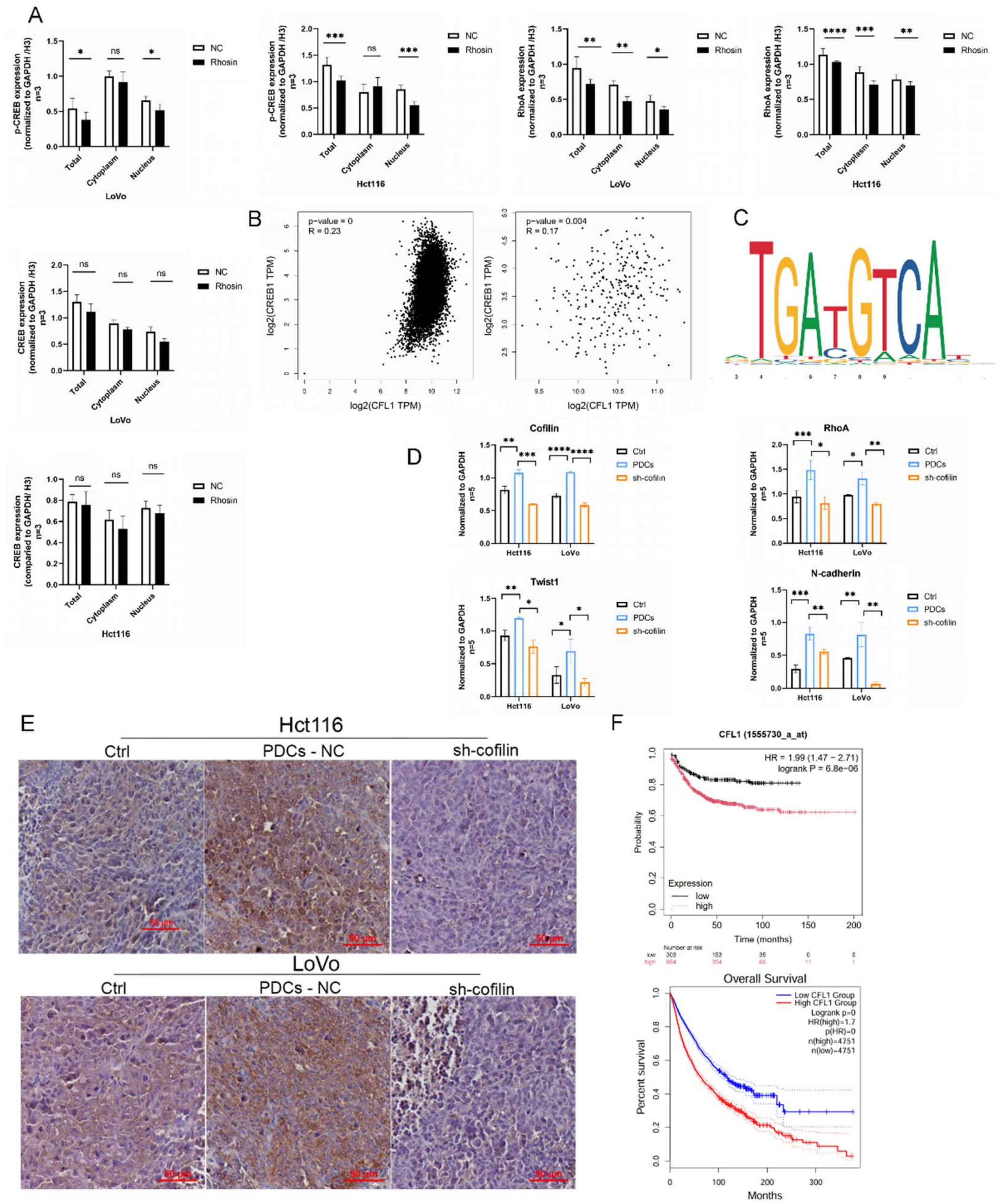

Fig. S5. A. Statistical analysis of WB results showing total, cytoplasmic, and nuclear RhoA, CREB, and p-CREB expression after treatment with 20 μM Rhosin in Hct116 and LoVo PDCs. Comparisons between pairs of groups were performed using an unpaired two-tailed Welch's t-test. B. GEPIA database analysis showing the correlation between cofilin and CREB mRNA levels. C. JASPAR database prediction of CREB-binding sites in the cofilin promoter region. D. Statistical analysis of WB results showing cofilin, RhoA, N-cadherin, and Twist1 expression in control, PDC-NC, and sh-cofilin groups in Hct116 and LoVo xenograft tumors. Statistical significance was determined using the Kruskal-Wallis' test followed by post-hoc analysis with Bonferroni correction. E. IHC staining of cofilin in Hct116 and LoVo xenograft tumors from the control, PDCs, and sh-cofilin groups. F. Kaplan-Meier plotter database analysis showing the association between OS and cofilin expression.

Ctrl, control cells; PDC-NC, negative control of PDCs; sh-cofilin, knockdown of cofilin in PDCs; *$P<0.05$, **$P<0.01$, ***$P<0.001$, ****$P<0.0001$; n.s., not significant.
